# Unveiling Systemic Biomarkers and Metabolic Mechanisms in Glaucoma Progression from Multi-Omics Insights

**DOI:** 10.3390/ijms27062848

**Published:** 2026-03-21

**Authors:** Shengshu Sun, Ning Xu, Ge Bai, Youhan Ao, An Wang, Jiaying Sun, Yifei Huang, Liqiang Wang

**Affiliations:** 1 Senior Department of Ophthalmology, The Third Medical Center of Chinese PLA General Hospital, Medical School of Chinese People’s Liberation Army, Beijing 100853, China; sshengshuamy@163.com (S.S.); 16601558976@163.com (N.X.); 18790561137@163.com (G.B.); 301yk@sina.com (Y.H.); 2 School of Medicine, Nankai University, Tianjin 300071, China; ayh071025@163.com

**Keywords:** glaucoma, metabolomics, transcriptome, mendelian randomization, blood and urine biomarkers, validation analysis

## Abstract

Early diagnosis of glaucoma remains challenging due to its asymptomatic onset and multifactorial pathological mechanisms. Growing evidence indicates that metabolic disorders and systemic molecular alterations play significant roles in glaucoma pathogenesis. However, reliable biomarkers and corresponding specific mechanisms remain unclear. In this study, we employed a multi-omics approach that encompassed metabolomics, transcriptomics, and Mendelian randomization to investigate the association between glaucoma and 35 types of blood and urine biomarkers. Metabolic pathway analysis was conducted using pathway enrichment analysis of differentially expressed genes based on the Kyoto Encyclopedia of Genes and Genomes (KEGG) database. Our study indicated that glaucoma contributed to elevated calcium concentration (OR = 1.044, 95% CI: 1.002–1.088, *p* = 0.039) in blood and urine, mediated by cell membrane calcium channels and calcium release from intracellular storage. Conversely, glucose was found to contribute to high glaucoma risk (OR = 1.324, 95% CI: 1.143–1.533, *p* = 0.0002), mediated by increased aqueous humor production, elevated intraocular pressure, endoplasmic reticulum stress, and oxidative stress. Validation experiments showed that calcium levels in blood, urine, and retina were elevated in the glaucoma group, and elevated glucose levels significantly reduced the 661W cell viability and induced apoptosis. This study offers new insights into the specific mechanisms linking blood and urine biomarkers to glaucoma, contributing to its prevention and screening.

## 1. Introduction

Glaucoma is characterized by elevated intraocular pressure (IOP) and the progressive loss of retinal ganglion cells (RGCs), causing retinal neurodegeneration and irreversible blindness, affecting over 95 million individuals worldwide [1,2,3]. Elevated IOP and damaged trabecular meshwork are widely recognized as the primary causes of primary glaucoma [4,5]. However, the etiology of glaucoma is multifactorial, and the precise sequence of pathological events and the key factors driving RGC degeneration remain poorly understood. In addition, due to the insidious onset and slow progression, early diagnosis and intervention are challenging [6,7]. Consequently, there is an urgent need to uncover the underlying mechanisms, explore new diagnostic methods, and develop effective therapies to prevent and treat glaucomatous neurodegeneration.

Accumulating evidence suggests that glaucoma arises from genetic and environmental influences. Metabolomics has been proven effective in diagnosing, classifying, and monitoring disease progression by analyzing metabolic byproducts, presenting a promising approach to the identification of disease-specific biomarkers [8,9,10]. Studies suggest that changes in metabolite levels in serum, aqueous humor, and tear fluid may serve as potential candidate biomarkers for glaucoma, including amino acids, proteins, lipids, and other metabolites [11,12,13,14]. For instance, one study reported significant enrichment of glutathione metabolism, glyoxylate and dicarboxylic acid metabolism, and biotin metabolism in serum samples from glaucoma patients [15]. In addition, biomarker screening in blood and urine is a common and suitable choice for clinical applications because they are easily accessible. Some systemic or genetic factors influencing glaucoma risk may be reflected through biomarkers in blood and urine, making analyses of these biofluids valuable for risk assessment [16,17,18,19]. However, there is a lack of research on the relationship between biomarkers and glaucoma progression. Additional biomarkers with stronger associations may exist but have yet to be identified and require further validation through genetic analysis and biological experiments.

Mendelian randomization (MR) is an analytic approach that employs genetic variations to assess the relationship between outcomes and risk factors. By investigating the random distribution of genetic variations across populations, MR can effectively avoid the confounding biases that may commonly arise in traditional observational studies, providing a more reliable and robust means of testing causal relationships [20]. As such, MR offers a valuable and reliable tool for understanding the causal effects of various risk factors on diseases, including those that are difficult to investigate through conventional research methods. With the advances of disease-associated single-nucleotide polymorphisms (SNPs) uncovered by genome-wide association studies (GWASs), MR has achieved increasing application fields. Though there are studies suggesting associations between metabolites and glaucoma [21,22], there is a lack of deep understanding of a wider range of biomarkers [23,24].

**Figure 1:**
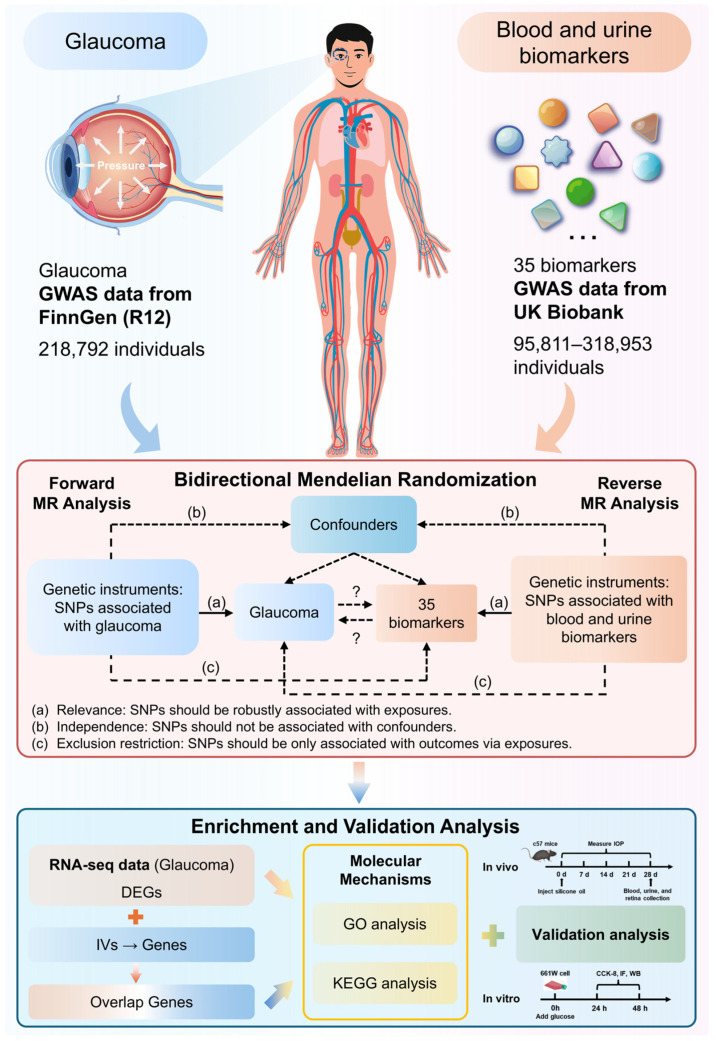
Flow chart of the overall study design.

Aiming to explore sophisticated underlying molecular mechanisms between blood and urine biomarkers and glaucoma, this study employed a multi-omics approach integrating metabolomics, transcriptomics, and a bidirectional MR, as shown in Figure 1. This study not only screens out blood and urine biomarkers related to glaucoma at the genetic level but also maps the associated metabolisms and uncovers transcriptional mechanisms.

## 2. Results

### 2.1. Forward MR Analysis: The Causal Effects of Glaucoma on Biomarkers

The Manhattan plot (Figure S1A) illustrated the distribution of SNPs across the chromosome by −log10 (*p*-value), presenting the genome-wide association results for glaucoma and highlighting the SNPs with a significance threshold (*p* < 5 × 10^−8^). The information from the GWAS datasets is summarized in Table S1. A total of nine SNPs with glaucoma were selected as instrumental variables (IVs) based on the selection criteria, which were identified from GWAS with genome-wide significance, linkage disequilibrium (LD) independence (r^2^ < 0.001, within a 10 Mb window), and F-statistic threshold (F > 10), thereby mitigating the potential for weak instrument bias. The causal effects of glaucoma on 35 blood and urine biomarkers were assessed using five MR analysis methods, as shown in Table S2.

**Figure 2:**
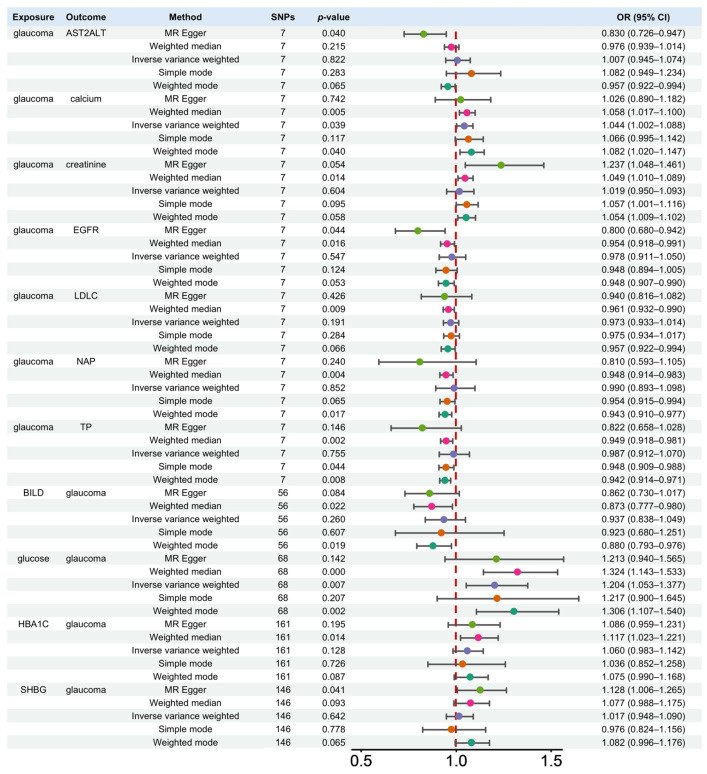
Causal effects of bidirectional MR analysis between glaucoma and blood and urine biomarkers using five MR analysis methods.

**Figure 3:**
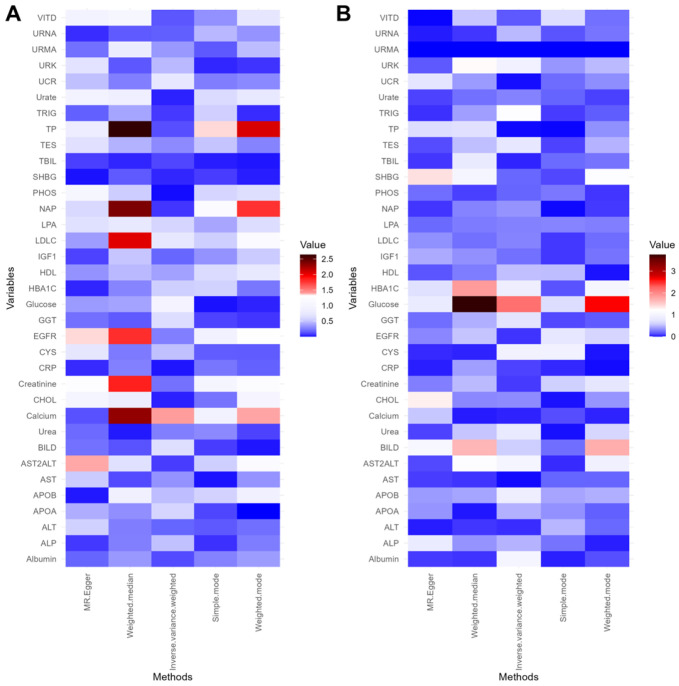
MR analysis results of −log10 (*p*-value) using five methods. (**A**) Heat map of forward MR analysis. (**B**) Heat map of reverse MR analysis.

Among 35 biomarkers, 7 biomarkers were found to be associated with the presence of glaucoma in these analysis methods (Figure 2 and Figure 3A). Remarkably, glaucoma contributed to elevated calcium levels through three methods, including inverse-variance weighted (IVW) (OR = 1.044, 95% CI: 1.002–1.088, *p* = 0.039), weighted median (OR = 1.058, 95% CI: 1.017–1.1, *p* = 0.0049), and weighted mode (OR = 1.082, 95% CI: 1.02–1.147, *p* = 0.04). In addition, the weighted median method indicated that glaucoma contributed to elevated creatinine (OR = 1.049, 95% CI: 1.049–1.010, *p* = 0.0138) levels. However, it resulted in a decrease in the levels of estimated glomerular filtration rate (EGFR, OR = 0.954, 95% CI: 0.918–0.991, *p* = 0.0162), LDL cholesterol (LDLC, OR = 0.961, 95% CI: 0.932–0.99, *p* = 0.0091), non-albumin protein (NAP, OR = 0.948, 95% CI: 0.914–0.983, *p* = 0.0041), and total protein (TP, OR = 0.949, 95% CI: 0.918–0.981, *p* = 0.0023). Furthermore, the MR Egger method indicated that glaucoma contributed to the decrease in the aspartate aminotransferase (AST)–alanine aminotransferase (ALT) ratio (AST2ALT, OR = 0.830, 95% CI: 0.726–0.947, *p* = 0.04).

**Figure 4:**
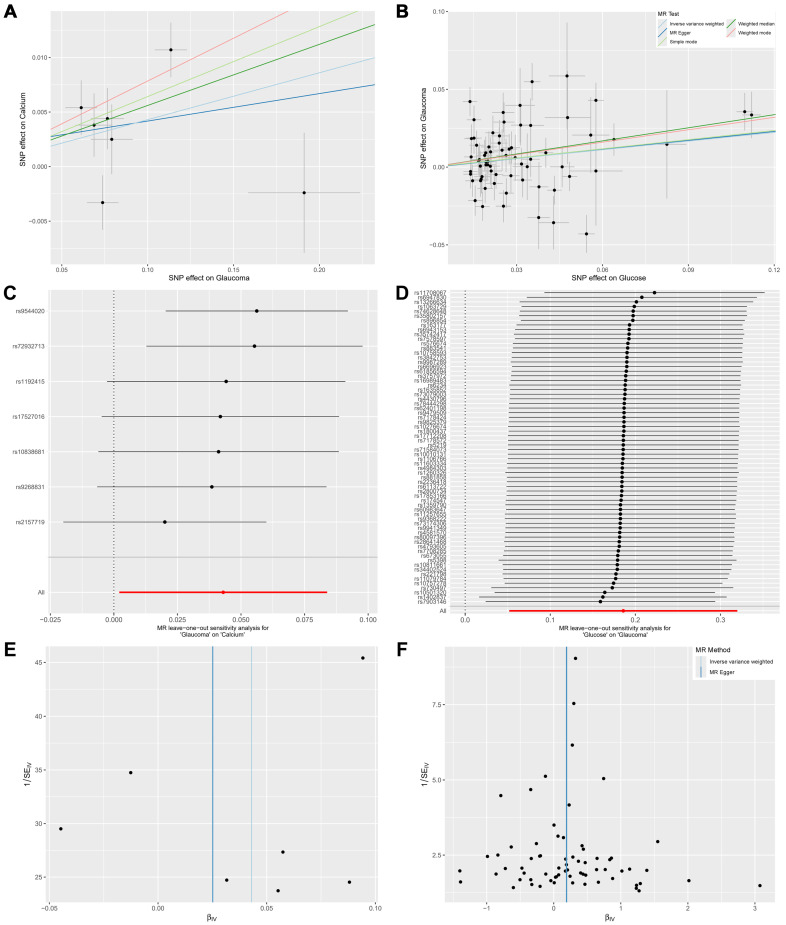
Sensitivity analysis of MR results. (**A**) SNP effect of glaucoma on calcium. (**B**) SNP effect of glucose on glaucoma. (**C**) Leave-one-out analysis of glaucoma on calcium. (**D**) Leave-one-out analysis of glucose on glaucoma. (**E**) Funnel plot of the effect of glaucoma on calcium. (**F**) Funnel plot of the effect of glucose on glaucoma.

The scatter plot (Figure 4A and Figure S2) showed the association between the SNP effect on glaucoma (X-axis) and the SNP effect on biomarkers (Y-axis). Positive linear relationships were observed from two biomarkers containing calcium and creatinine. Five biomarkers, including AST2ALT, EGFR, LDLC, NAP, and TP, presented negative linear relationships with glaucoma.

### 2.2. Reverse MR Analysis: The Causal Effects of Biomarkers on Glaucoma

SNPs from blood and urine biomarkers were selected as IVs based on the selection criteria to assess the causal effects on glaucoma using five MR analysis methods. The MR results of the effect of 35 blood and urine biomarkers on glaucoma are shown in Table S3. Four biomarkers were identified as having a significant contribution to the development of glaucoma by five MR analysis methods, with their effects either positively or negatively influencing glaucoma (Figure 2 and Figure 3B). Notably, the analysis result demonstrated that glucose played a promotional role in glaucoma progression through two methods, including IVW (OR = 1.204, 95% CI: 1.053–1.377, *p* = 0.007) and weighted median (OR = 1.324, 95% CI: 1.143–1.533, *p* = 0.0002). In addition, the weighted median method suggested that direct bilirubin (BILD) played a protective role in glaucoma development (OR = 0.873, 95% CI: 0.777–0.980, *p* = 0.0216), while hemoglobin A1c (HBA1C, OR = 1.117, 95% CI: 1.023–1.221, *p* = 0.0138) exerted a promotional effect on glaucoma development. In addition, the MR Egger method indicated that sex hormone-binding globulin (SHBG, OR = 1.128, 95% CI: 1.006–1.265, *p* = 0.0408) contributed to the onset of glaucoma.

The scatter plot of five MR methods presented the SNP effect of biomarkers on glaucoma (Figure 4B and Figure S3). Negative linear relationships were observed from BILD on glaucoma. Three biomarkers, including glucose, HBA1C, and SHBG, presented positive linear relationships with glaucoma.

### 2.3. Sensitivity Analyses

Sensitivity analyses were conducted for MR results with *p* < 0.05, including the effect of seven biomarkers on glaucoma in the forward MR and the effect of glaucoma on four biomarkers in the reverse MR. The leave-one-out analysis was performed to assess the robustness of the bidirectional MR results (Figure 4C,D, Figures S4 and S5). There was no significant change in causal effects by excluding individual SNPs, indicating that the results were robust and not affected by any single genetic variant.

Pleiotropy was assessed using the regression intercept from the MR-Egger and MR-IVW methods, while Cochran’s Q test was applied to evaluate global heterogeneity among the genetic instruments. Heterogeneity among the associations between urine and glaucoma was observed according to Cochran’s Q test (I^2^ > 25% and *p* < 0.05) and *p* < 0.05 for the MR-Egger and MR-IVW test (Table 1). Funnel plots (Figure 4E,F, Figures S6 and S7) from the MR-Egger method indicated SNP bias in the effect of glaucoma on biomarkers, whereas the IVW method showed no bias. No bias was observed in the effect of biomarkers on glaucoma. To address the observed heterogeneity, we performed an MR-PRESSO analysis to identify potential outliers. After excluding outlier SNPs using the distortion test, the heterogeneity test was no longer significant (*p* > 0.05), suggesting that the detected heterogeneity was largely driven by these outliers (Table 2). Additionally, no evidence for directional pleiotropy was found for effects of glaucoma on calcium, creatinine, EGFR, LDLC, NAP, and TP, as well as effects of BILD, glucose, and HBA1C on glaucoma (Table 1).

### 2.4. Enrichment Investigation

**Figure 5:**
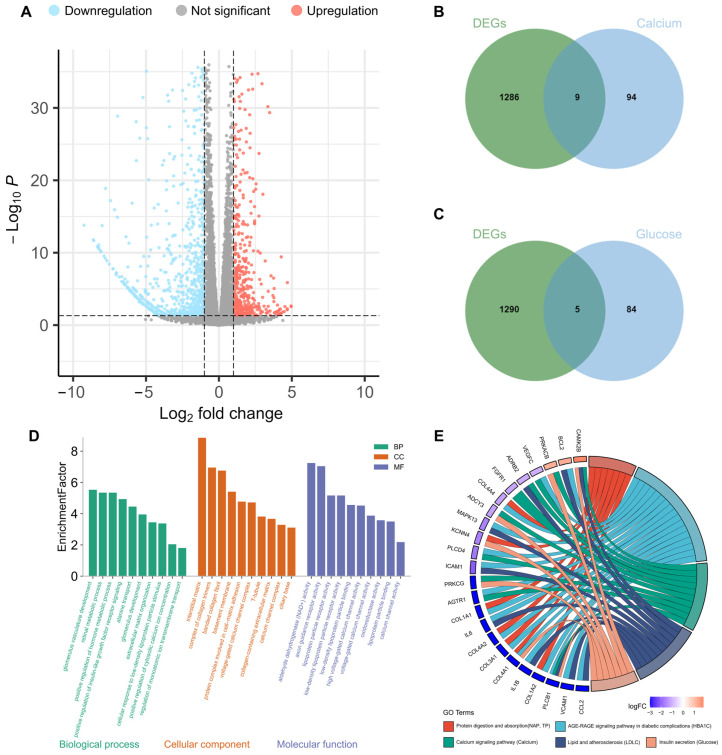
Enrichment analysis. (**A**) DEGs in the GSE216660 dataset (Glaucoma vs. controls) (**B**) Venn diagram between DEGs and calcium-associated genes. (**C**) Venn diagram between DEGs and glucose-associated genes. (**D**) GO enrichment analysis of trabecular meshwork cells of healthy and glaucoma patients. (**E**) KEGG enrichment analysis of trabecular meshwork cells of healthy and glaucoma patients.

Based on the MR analysis results, the primary method, IVW, demonstrated the causal effects of glaucoma on calcium and glucose on glaucoma. To explore the molecular mechanisms of glaucoma, we performed Gene Ontology (GO) and Kyoto Encyclopedia of Genes and Genomes (KEGG) enrichment analyses to identify shared functions and associated pathways among genes. As shown in the volcano plot Figure 5A, 1295 differentially expressed genes (DEGs) were identified based on a threshold on |log_2_FC| > 1 and *p* < 0.05 through transcriptomic profiling of the Gene Expression Omnibus (GEO) dataset GSE216660, including 449 upregulated and 846 downregulated genes. GO analysis confirmed that calcium channel activity, positive regulation of insulin-like growth factor receptor signaling, and lipoprotein particle receptor activity were primarily enriched (Figure 5D). KEGG analysis identified the calcium signaling pathway, insulin secretion, and AGE-RAGE signaling pathways as the major mechanisms (Figure 5E).

### 2.5. Identification of Overlapping Genes

**Figure 6:**
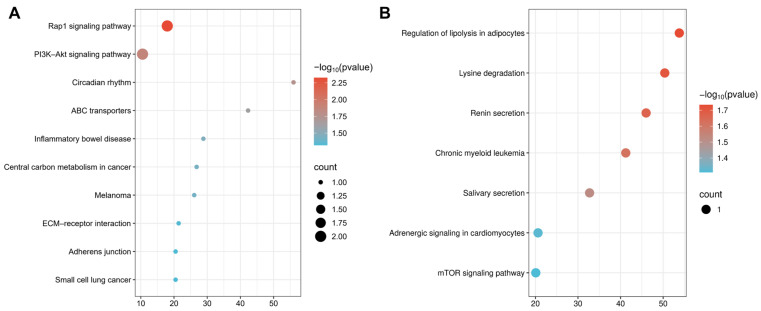
Functional analysis of overlapping genes between DEGs in glaucoma and biomarker-associated genes. (**A**) KEGG pathway enrichment analysis for overlapping genes with calcium. (**B**) KEGG pathway enrichment analysis for overlapping genes with glucose.

Biomarker-associated genes were obtained by querying the online database SNPnexus, including 103 genes for calcium and 89 genes for glucose. To identify overlapping genes between glaucoma DEGs and biomarker-associated genes, Venn diagrams were generated (Figure 5B,C), showing nine overlapping genes between DEGs and calcium-associated genes and five overlapping genes between DEGs and glucose-associated genes. To deeply explore the biological mechanisms between biomarker-associated genes and DEGs, KEGG enrichment analyses were conducted using overlapping genes. By analyzing the calcium-associated genes, KEGG analysis highlighted key molecular mechanisms, including the Rap1 signaling pathway, the PI3K-Akt signaling pathway, ABC transporters, etc. (Figure 6A). In addition, KEGG analysis using glucose-associated genes revealed the significant involvement in molecular mechanisms, including the mTOR signaling pathway, Renin secretion, Adrenergic signaling in cardiomyocytes, etc. (Figure 6B).

### 2.6. Validation Investigation

**Figure 7:**
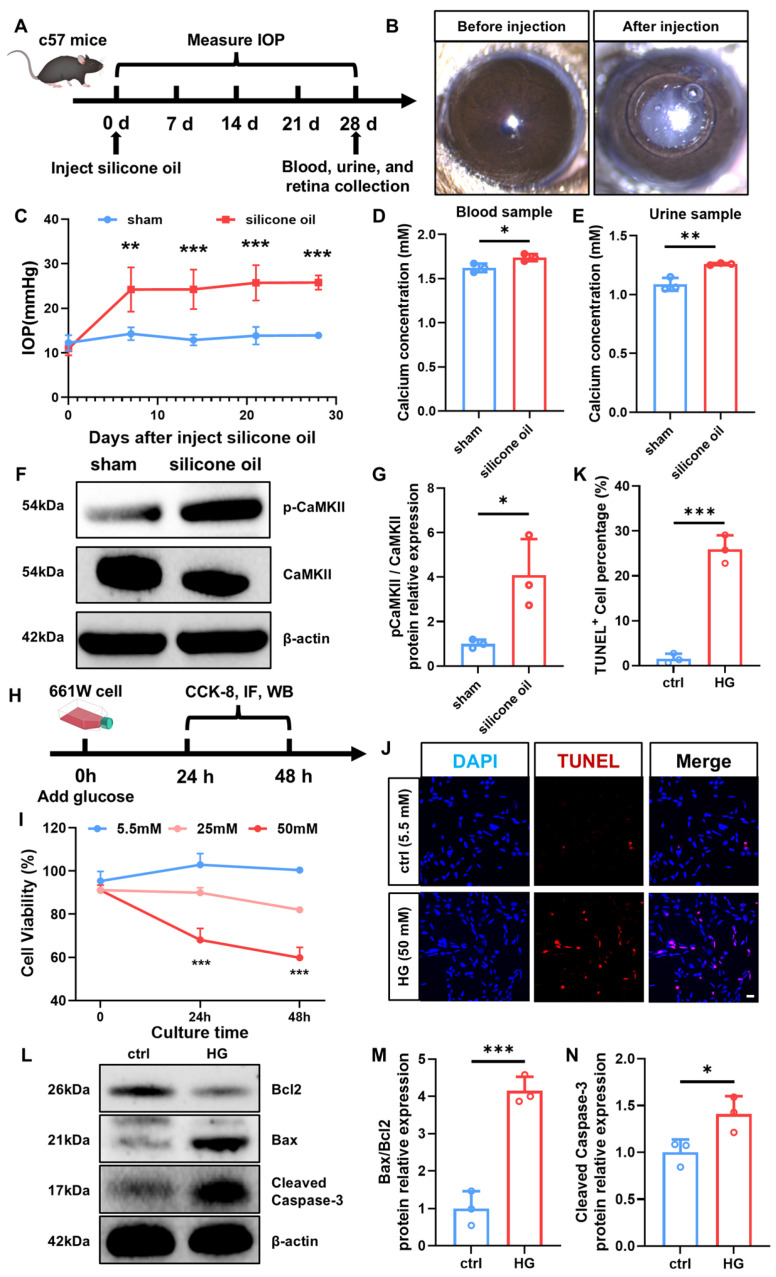
Validation analysis of calcium in vivo and glucose in vitro. (**A**) Inject silicone oil into the anterior chamber of C57 mice, measure IOP every 7 days, and collect blood and urine samples on day 28. (**B**) Anterior segment photographs before and after silicone oil injection. (**C**) Quantitative analysis of IOP in the sham and silicone oil groups (n = 3/group). (**D**) Quantitative analysis of calcium concentration of the sham and silicone oil groups in blood (n = 3/group). (**E**) Quantitative analysis of calcium concentration of the sham and silicone oil groups in urine (n = 3/group). (**F**) Protein expression levels of the p-CaMKII and CaMKII in the retina in the sham and silicone oil groups (n = 3/group). (**G**) Quantitative analysis of the level of p-CaMKII/CaMKII proteins (n = 3/group). (**H**) The schematic diagram of the experimental design for the ctrl and HG groups. (**I**) The cell viability investigation of ctrl and HG groups by using the CCK8 assay (n = 3/group). (**J**) TUNEL immunofluorescence images of 661W cells in the ctrl and HG groups after 48h of culture, scale bar: 20 μm. (**K**) Quantitative analysis of TUNEL+ 661W cells (n = 3/group). (**L**) Protein expression levels of the apoptosis markers Bcl2, Bax, and Cleaved Caspase-3 in 661W cells from the ctrl and HG groups after 48h of culture. (**M**) Quantitative analysis of the level of Bax/Bcl2 proteins (n = 3/group). (**N**) Quantitative analysis of the level of Cleaved Caspase-3 protein (n = 3/group). (* *p* < 0.05, ** *p* < 0.01, and *** *p* <0.001).

The validation analysis was performed based on C57 mice and 661W cells. The high degree of conservation in glaucoma-related phenotypes between human and mouse eyes supports the use of mice as an appropriate and informative glaucoma model for elucidating the molecular pathways [25]. Briefly, based on a previous study [26], we successfully established a glaucoma model in C57 mice by injecting silicone oil into the anterior chamber (Figure 7A). Anterior segment photography revealed complete coverage of the pupil by silicone oil, with significantly elevated IOP at various time points after injection (Figure 7B,C). On day 28 after silicone oil injection, calcium concentrations were elevated in both blood and urine samples from the C57 mice (Figure 7D,E). In addition, the level of p-CaMKII in the retina increased (Figure 7F,G).

Irreversible damage to RGCs is one of the core pathological mechanisms through which glaucoma impairs visual function. To investigate whether HG influences glaucoma-related RGC damage, as described in previous studies [27], 661W cells, a mouse immortalized cell line with features of retinal ganglion precursor-like cells, were selected to establish an HG model (Figure 7H). The CCK8 assay results showed a significant reduction in cell viability in the HG group compared with the control group at different time points (Figure 7I), suggesting that HG might inhibit 661W cell viability. Moreover, results demonstrated a marked increase in TUNEL-positive cells in the HG group after 48 h of culture (Figure 7J,K). In addition, WB analysis suggested a significant increase in the apoptosis-related protein Cleaved Caspase-3 and Bax/Bcl2 ratio in the HG group (Figure 7L–N).

## 3. Discussion

To our best knowledge, this study first investigated the relationship and underlying molecular mechanisms between glaucoma and blood and urine biomarkers by integrating multi-omics enrichment analyses and MR approaches. Furthermore, this relationship between blood and urine biomarkers and glaucoma was demonstrated in vivo and in vitro experiments. In this study, we confirmed that glaucoma exerts a causal effect on AST2ALT, calcium, creatinine, EGFR, LDLC, NAP, and TP in blood and urine biomarkers. Conversely, biomarkers BILD, glucose, HBA1C, and SHBG were found to contribute to the progression of glaucoma, either promoting or inhibiting its course. Among these, the primary method IVW demonstrated the causal effect of glaucoma on calcium and glucose on glaucoma. Enrichment analysis revealed a bidirectional relationship between glaucoma and biomarkers. Specifically, glaucoma may influence biomarker secretion through the regulation of molecular mechanisms and involvement in biological processes, while other biomarkers may contribute to glaucoma progression by participating in key molecular pathways. Validation experiments demonstrated elevated calcium levels in blood and urine samples from the glaucoma group, while hyperglycemia in these samples may contribute to the onset of glaucoma. This work not only deepens the understanding of the pathophysiological mechanisms of glaucoma but also lays a scientific foundation for identifying susceptible populations and conducting targeted intervention studies in clinical practice.

Our study indicated that glaucoma contributed to higher concentrations of calcium in blood and urine, supported by enrichment analysis and validation experiments. Prior cohort studies have demonstrated that glaucoma patients may exhibit calcium channel abnormalities, which can disrupt calcium homeostasis in retinal neurons and may be reflected by altered calcium levels in blood or urine [28,29]. These results are consistent with our findings and further support the conclusion that glaucoma is involved in alterations of calcium levels in blood and urine. Mechanistically, as a ubiquitous second messenger, calcium participates in regulating multiple physiological processes and is implicated in various diseases, including cancer, fibrosis, and glaucoma. Studies have revealed the presence of oxidative stress, elevated calcium channel expression, enhanced calcium-dependent pump and exchanger activity, and abnormal cytoplasmic calcium level increases in human glaucoma trabecular meshwork fibroblasts [30]. Enrichment analysis in this study suggested that this elevation was mediated by cell membrane calcium channels and involved calcium release from intracellular storage compartments in the endoplasmic reticulum and mitochondria. Specifically, through KEGG analysis of overlapping genes, glaucoma may increase calcium concentrations in blood and urine through multiple signaling pathways. Under pathological conditions, the Rap1 signaling pathway alters the stability of the aqueous humor and retinal vascular barriers, leading to increased local membrane calcium permeability. Meanwhile, the PI3K-Akt signaling pathway regulates intracellular calcium storage and channel activity, promoting the release of calcium into the extracellular space. ABC transporters may export excess calcium and calcium-associated complexes into the blood and urine, acting as ATP-dependent efflux pumps. The synergistic effect of these pathways may collectively explain the molecular mechanisms underlying the elevated calcium-related biomarkers observed in the blood and urine of patients with glaucoma. Previous studies have demonstrated a close association between glaucoma and calcium dysregulation, as well as other central nervous system disorders, suggesting that aberrant calcium activation contributes to RGC death following injury [31,32,33,34]. As a central coordinator and effector of calcium signaling, CaMKII mediates neuroprotection in glaucoma models through reactivation of the CaMKII-CREB pathway, thereby effectively protecting RGCs [31,32]. In central nervous system diseases, microglia modulate immune functions by regulating intracellular calcium signaling. Calcium dysregulation is closely linked to microglial activation, where the calcium-sensing receptor promotes NLRP3 inflammasome activation by increasing cytosolic calcium levels [35,36]. Furthermore, the PKC pathway serves as a critical downstream effector of calcium, transducing calcium signals into specific functional alterations that underline the dynamic response of microglia. In glaucoma, specific patterns of intracellular calcium fluctuations drive microglial phenotypic shifts. This process relies on calcium signaling cascades to activate the transcription factor NF-κB, triggering the massive synthesis and release of pro-inflammatory mediators such as TNF-α, which ultimately modulates the retinal inflammatory microenvironment [37]. In vivo experiments revealed elevated calcium levels in blood and urine samples from glaucoma mice, with a more pronounced increase observed in urine. In addition, the increased expression of p-CaMKII reflected disrupted calcium signaling and elevated calcium levels in the retina. These findings provide a basis for predicting glaucoma through alterations in calcium levels in systemic circulation and ocular tissues.

Our study identified elevated levels of glucose as risk factors for the onset of glaucoma. Studies have consistently confirmed that diabetes is a significant risk factor for glaucoma [38,39,40]. These findings are consistent with the results of our study, indicating that elevated glucose levels contribute to the high glaucoma risk, and are also supported by several other studies [41,42,43]. Enrichment analysis suggested that hyperglycemia can increase the risk of glaucoma through multiple molecular mechanisms. A hyperglycemic state can activate sympathetic signaling (adrenergic signaling) and renin secretion, leading to increased aqueous humor production and elevated IOP. Meanwhile, high glucose levels activate the mTOR pathway, inducing endoplasmic reticulum stress, oxidative stress, and retinal neuronal damage, thereby further accelerating the progression of glaucoma. The coordinated effects of these pathways provide a molecular explanation for the increased incidence of glaucoma in patients with diabetes. Furthermore, in vitro experiments revealed that 661W cells in HG culture medium exhibited significantly reduced viability and increased apoptosis levels, suggesting that elevated glucose levels may contribute to the increased risk of glaucoma. Studies have demonstrated the contribution of diabetes to an increased risk of glaucoma [41,42,43]. A study demonstrated elevated IOP and blood glucose levels during glaucoma progression. Meanwhile, the activation of the mTOR pathway contributed to metabolic pathway disruption and glaucoma progression [41]. Moreover, another study concluded that prolonged exposure to elevated blood glucose levels resulted in mitochondrial DNA damage of RGCs, thereby promoting glaucoma development [42].

In addition to glucose, our study found a positive correlation between the risk of glaucoma and HBA1C levels, which reflected diabetes control and the average glucose levels over a longer period. The result was consistent with prior studies that implicated HBA1C levels in the pathophysiology of glaucoma [43,44]. The underlying mechanisms were thought to involve the impact of elevated blood glucose levels on ocular blood flow, increased oxidative stress, and inflammatory pathways, all of which may contribute to the development and progression of glaucoma. Additionally, hyperglycemia-induced retinal ischemia and hypoxia, followed by the release of angiogenic factors, is another cause of glaucoma. Ischemic conditions and the release of angiogenic factors promote iris neovascularization and fibrovascular membrane proliferation in the anterior chamber angle. These changes obstruct the trabecular meshwork, leading to peripheral anterior synechiae and progressive angle closure.

Furthermore, glaucoma showed a causal relationship with other blood and urine biomarkers. Previous studies have demonstrated a significant association between glaucoma and chronic kidney disease (CKD), showing that glaucoma patients were over three times more likely to develop CKD than those without glaucoma [45]. Potential mechanisms underlying this association include confounding effects of shared comorbidities such as microvascular dysfunction and ischemia, renin–angiotensin system dysfunction, oxidative stress, and inflammatory responses [45]. Creatinine, EGFR, NAP, and TP are urine biomarkers commonly used in clinical practice to assess renal function. Creatinine is a product of reactions involving creatine kinase and adenosine triphosphate and plays a key role in energy supply for muscles, including those in the ciliary body [46]. Previous studies have confirmed elevated creatinine concentrations in the aqueous humor of glaucoma patients, further supporting its potential role in the disease’s metabolomics [47,48,49]. Evidence has shown elevated aqueous humor creatinine concentration in glaucoma patients, suggesting that the creatinine may be involved in the metabolic alterations associated with glaucoma [49]. EGFR reflects the kidney’s filtration capacity and is a key indicator of renal function. In this study, glaucoma was found to negatively impact EGFR levels, indicating an association with CKD that may stem from shared etiologies and pathophysiological mechanisms [45,50]. In the genetics study, the non-albumin protein (NAP) level was defined as the difference between total protein (TP) and albumin levels, which serve as biomarkers from kidney tissue [51]. Few studies have investigated the relationship between TP, NAP, and glaucoma. In this study, glaucoma-associated SNPs showed a negative correlation with TP and NAP levels. Under glaucoma conditions, elevated IOP may affect ocular hemodynamics, subsequently impacting systemic blood circulation, which can lead to microvascular damage, including in the kidney, thereby reducing the kidney’s ability to excrete proteins [52,53,54]. Moreover, the oxidative stress associated with both glaucoma and previously mentioned CKD may lead to lipid peroxidation, which could result in decreased TP and NAP levels [55,56].

AST and ALT are common markers of liver function, and their ratio (AST2ALT) is often used to assess liver health. One study suggested that the AST2ALT ratio was a risk factor for a large vertical cup-to-disc ratio, which was associated with glaucoma [57]. Results in this study showed that glaucoma may account for the lower ratio of AST2ALT. However, the significant p-value for pleiotropy in this study suggested that other factors may influence the AST2ALT ratio, which could reflect the indirect impact of glaucoma on systemic metabolism, particularly through chronic inflammation, oxidative stress, and interactions with other diseases [58,59,60].

Recent epidemiological studies suggested a potential link between blood lipid levels and glaucoma, although the findings were inconsistent [61,62]. This study indicated a weak negative correlation between LDLC levels and glaucoma progression, supported by a cohort study [61]. In contrast, a cross-sectional study reported a positive correlation between high serum LDLC levels and glaucoma [62]. This indicates that the relationship between LDLC and glaucoma is complex and influenced by multiple factors, necessitating more in-depth analyses incorporating additional biomarkers and larger genetic datasets.

BILD is a byproduct of red blood cell breakdown, processed in the liver where it is conjugated with glucuronic acid and excreted in bile. It possesses potent antioxidant properties and may serve as a serum biomarker for disorders associated with oxidative stress and inflammation [63]. The results of this study suggested that elevated BILD levels may confer a protective effect against glaucoma, likely owing to its antioxidant properties that help mitigate oxidative stress associated with glaucoma [64]. A cross-sectional case–control study supported that the low total bilirubin level was a protective factor for primary open-angle glaucoma (POAG) [65]. In addition, a retrospective case–control study and an MR study indicated that the BILD levels were lower in glaucoma patients compared to controls, suggesting that these antioxidants may contribute to glaucoma treatment by depleting them [66,67]. Our study demonstrated an association between SHBG levels and the risk of glaucoma. However, this relationship was influenced by pleiotropy. One previous study observed no significant associations between SHBG and POAG risk in postmenopausal women. Further research is needed to more clearly understand the mechanisms underlying this association.

The MR results indicated that the heterogeneity test yielded *p* < 0.05, showing the presence of heterogeneity. However, the heterogeneity test was no longer significant (*p* > 0.05) after excluding the potential outliers identified by the distortion test. In addition, no directional pleiotropy or bias was detected using the IVW method, and the leave-one-out analysis demonstrated the stability of the causal effects. These findings indicated that the causal estimates were robust, as supported by the consistency of effect directions across multiple MR methods and sensitivity analyses.

The primary strengths of this study lie in its comprehensive application of a multi-omics approach, integrating metabolomics, transcriptome, and MR, complemented by biological validation experiments. By employing these investigation approaches, the validity and credibility of this study are enhanced. This work bridges the gap between causal inference and functional mechanisms, providing new insights into the underlying mechanisms and potential biomarkers for early diagnosis and intervention of glaucoma. However, several limitations in this study should be acknowledged. First, this study is limited by the datasets containing only individuals of European ancestry, due to the unavailability of GWAS summary statistics for other populations. Therefore, the generalizability of these findings beyond European populations should be interpreted with caution. Further studies that encompass diverse populations with well-characterized demographic and genetic profiles will be essential for improving the generalizability of these findings. In addition, this study focused on the relationship between glaucoma and systemic metabolite levels. Considering the expression disparities between systemic metabolite levels and those within ocular tissues, future studies should focus on systematically comparing systemic metabolite profiles with their corresponding concentrations in visual organs, particularly at disease-relevant pathological sites. Furthermore, systemic metabolite levels are modulated by a range of individual-specific factors, including comorbidities, diet, and diverse occupational and environmental factors. Therefore, further studies should consider and classify these individual-specific factors to obtain a comprehensive understanding. Nevertheless, this study relied on a mouse model of glaucoma to investigate alterations in calcium-related expression, which requires further validation in human cohorts. This approach may offer valuable insights into the complex mechanisms underlying glaucoma development.

## 4. Materials and Methods

### 4.1. Study Design

To investigate the relationship between glaucoma and blood and urine biomarkers, the MR and multi-omics (metabolomics and transcriptomics) approaches were performed with validation experiments. Figure 1 presents an overall diagram of the study design. A bidirectional MR approach was conducted to systematically evaluate the causal relationship between blood and urine biomarkers and glaucoma. Furthermore, DEGs, biomarker-associated genes, and the overlapping genes were employed to perform enrichment analyses to explore potential molecular mechanisms. Finally, the relationship between glaucoma and biomarkers was validated through experiments. The multi-omics approach provides comprehensive validation of the relationship and functional mechanisms.

### 4.2. Data Sources

Datasets for blood and urine biomarkers were obtained from the genetic study, which included 363,228 individuals in the UK Biobank (https://doi.org/10.35092/yhjc.12355382, accessed on 5 February 2025) [51]. This large-scale study analyzed numerous genetic variants associated with various biomarkers to investigate their genetic determinants and potential causal effects on diseases. The findings provide valuable insights into the genetic information of these biomarkers and their roles in disease development, offering a deeper understanding of their causal impact on disease risk. Additionally, the study highlights the potential for improving genetic risk stratification for common diseases through biomarker-based genetic analyses.

GWAS summary statistics for glaucoma were obtained from the FinnGen (R12 release), a study linking genomic data with digital healthcare records of Finnish participants aged 18 and older. The glaucoma GWAS included 218,792 individuals, comprising 8591 cases and 210,201 controls. Data are available and requested at the official websites (https://www.finngen.fi/fi, accessed on 5 February 2025). The samples used in the two-sample MR design were independent. Detailed information on the summary statistics included in the analysis is provided in Table S1. The GSE216660 dataset was sourced from the NCBI GEO database (https://www.ncbi.nlm.nih.gov/geo/, accessed on 12 November 2025).

### 4.3. Selection Criteria of Genetic Variants as Instrumental Variables

IVs in MR studies must satisfy three core assumptions: (i) relevance: the IV is strongly correlated with the exposure; (ii) exclusivity: the IV does not directly affect the outcome; and (iii) independence: the IV is not associated with any confounders.

SNPs achieving genome-wide significance (*p*-value < 5 × 10^−8^) were selected from the GWAS datasets and subjected to LD clumping using a threshold of r^2^ < 0.001 within a 10 Mb window, minimizing SNP correlations and preventing violations of the instrumental independence assumption. SNPs with a stronger correlation to the outcome than the exposure were excluded, as well as those showing notable influence in funnel and scatter plots. An F-statistic threshold of 10 was set to ensure robust correlations with exposure factors by excluding weak IVs.

### 4.4. Two-Sample Mendelian Randomization Methodology

The two-sample MR approach was employed to evaluate the total effect of individual exposure on an outcome using GWAS data of blood and urine biomarkers and glaucoma. Genetic associations were harmonized by aligning effect alleles across exposure and outcome datasets, without excluding palindromic variants. MR analyses were conducted using five methods, including weighted median, IVW, weighted mode, MR-Egger regression, and simple mode, to assess the mutual causal effect between glaucoma and blood and urine biomarkers. This study employed the IVW method for primary analysis.

To assess the validity of IVs and the robustness of MR analyses, several sensitivity analyses were conducted. The Cochran Q heterogeneity test was used to assess IV heterogeneity. MR-pleiotropy analysis was performed to identify potential pleiotropy with a threshold of *p*-value < 0.05. Additionally, the leave-one-out analysis was conducted by sequentially excluding each IV to examine the potential impact of individual IVs on the findings. Additionally, the MR-PRESSO global test was employed to identify outlier variants exhibiting horizontal pleiotropy. When significant outliers were identified, the MR-PRESSO distortion test was conducted to adjust the causal estimates, ensuring robustness. To assess the causal relationship of blood and urine biomarkers on glaucoma, the reverse MR analysis was employed to investigate the influence of biomarkers on glaucoma. Normal *p*-values were adjusted for multiple hypothesis testing using the Benja-mini-Hochberg method within each trait category. The R version 4.3.2 was employed for all analyses using the MendelianRandomization (version 0.10.0), TwoSampleMR (version 0.6.8), and MRPRESSO (version 1.0) packages.

### 4.5. Enrichment Analysis for IV-Associated Genes

DEGs between the glaucoma and control groups in the GSE216660 were identified using the DESeq2 (version 1.42.1) package with a threshold of |log_2_FC| > 1 and *p* < 0.05. To identify glaucoma-related metabolic pathways, GO and KEGG enrichment analyses were implemented using the cluster Profiler (version 4.10.1) package. This investigation aimed to determine whether the selected SNPs and IVs were associated with changes in the expression of specific genes, thereby allowing further speculation on whether these genes may play roles in the relationship between blood and urine biomarkers and glaucoma.

### 4.6. Identification and Enrichment Analysis of Overlapping Genes

To obtain the biomarker-associated genes, the SNPs were annotated to genes, including overlapped genes and nearest upstream and downstream genes. To explore the molecular mechanism between glaucoma and blood and urine biomarkers, the overlapping genes were identified between biomarker-associated genes and DEGs, which are key mediators in the underlying mechanisms. Subsequently, KEGG enrichment analysis was implemented using the cluster Profiler (version 4.10.1) package.

### 4.7. Validation Analysis

C57BL/6J (C57) wild-type mice, aged 6–8 weeks, were acquired from Beijing Spefo Laboratory Animal Technology (Beijing, China) and housed in a pathogen-free, temperature-controlled (22–24 °C) and humidity-controlled (40–70%) environment. To minimize pain and distress, all procedures were performed under appropriate anesthesia and analgesia. Glaucoma was induced in C57 mice using an established silicone oil model [26]. The silicone oil (2 μL) was injected into the anterior chamber to cover the pupil using a 33-G needle. The eyelids were closed to minimize silicone oil leakage. IOP was measured after dilating the eyes with a Tonometer on days 0, 7, 14, 21, and 28 after injection, and the blood, urine, and retina samples from the C57 mice were harvested for calcium concentration testing (Beyotime kit, S1063S, Shanghai, China) and Western blot analysis on day 28. No expected adverse events were observed throughout the duration of the study. The animal experiment protocol was reviewed and approved by the Institutional Animal Care and Use Committee (IACUC) of the Chinese People’s Liberation Army General Hospital.

To assess the influence of glucose on RGCs, cell experiments were conducted. Briefly, the 661W cells were cultured in high-glucose Dulbecco’s modified Eagle medium (DMEM, Beyotime, ST491), with 1% penicillin–streptomycin and 10% fetal bovine serum. To simulate a hyperglycemic environment, we designed a total of three groups: the control group (ctrl, 5.5 mM), the glucose group (25 mM), and the high-glucose group (HG, 50 mM). Cells were incubated for 48 h in a CO_2_ incubator at 37 °C with 5% CO_2._ Throughout this incubation, we performed a series of assays at various time points, including assessments of cell viability and apoptosis. The cell viability for each group was assessed with the CCK8 assay (Beyotime kit, C0038). Apoptotic cells were assessed using TUNEL staining (Beyotime kit, C1090) and Western blot analysis.

Each experiment was repeated three times, with results expressed as the mean ± standard deviation. Differences between groups were analyzed using the *t*-test or one-way analysis of variance followed by Tukey’s multiple comparison tests, considering *p* < 0.05 as the threshold for statistical significance.

## 5. Conclusions

In conclusion, this study provides supportive evidence and mechanistic insights for a causal relationship between glaucoma and various blood and urine biomarkers, including calcium, creatinine, EGFR, LDLC, NAP, TP, BILD, glucose, and HBA1C. Furthermore, enrichment analysis reveals molecular mechanisms and pathways for glaucoma-induced high calcium levels and the risk factor of glucose for glaucoma, substantiated through biological experiments. The findings of this study open vast prospects for the identification, mechanistic understanding, and risk assessment of biomarkers associated with glaucoma risk. Understanding the mechanisms by which blood and urine biomarkers function in glaucoma will provide a foundation for precision medicine and enhance the clinical management of glaucoma.

## Figures and Tables

**Table 1:** Summary of heterogeneity and pleiotropy for the significant results (*p* < 0.05) of the bidirectional MR analysis.

Exposure	Outcome	MR-IVW				MR-Egger				Pleiotropy	
		Q	Q_df	I^2^	Pval_Q	Q	Q_df	I^2^	Pval_Q	Intercept	*p*_Value
glaucoma	AST2ALT	46.908	6	87.209	1.95 × 10^−8^	16.625	5	69.924	5.27 × 10^−3^	1.81 × 10^−2^	2.95 × 10^−2^
glaucoma	calcium	17.388	6	65.494	7.96 × 10^−3^	17.158	5	70.859	4.21 × 10^−3^	1.65 × 10^−3^	8.06 × 10^−1^
glaucoma	creatinine	56.554	6	89.391	2.25 × 10^−10^	26.137	5	80.870	8.40 × 10^−5^	−1.81 × 10^−2^	6.07 × 10^−2^
glaucoma	EGFR	57.670	6	89.596	1.34 × 10^−10^	25.157	5	80.125	1.30 × 10^−4^	1.87 × 10^−2^	5.18 × 10^−2^
glaucoma	LDLC	19.641	6	69.451	3.21 × 10^−3^	18.681	5	73.235	2.20 × 10^−3^	3.21 × 10^−3^	6.34 × 10^−1^
glaucoma	NAP	111.746	6	94.631	8.78 × 10^−22^	82.321	5	93.926	2.74 × 10^−16^	1.87 × 10^−2^	2.39 × 10^−1^
glaucoma	TP	66.491	6	90.976	2.14 × 10^−12^	42.242	5	88.163	5.26 × 10^−8^	1.70 × 10^−2^	1.51 × 10^−1^
BILD	glaucoma	116.242	55	52.685	2.81 × 10^−6^	112.442	54	51.975	5.42 × 10^−6^	4.59× 10^−3^	1.82 × 10^−1^
glucose	glaucoma	173.413	67	61.364	2.25 × 10^−11^	173.401	66	61.938	1.38 × 10^−11^	−2.77 × 10^−4^	9.46 × 10^−1^
HBA1C	glaucoma	400.558	160	60.056	1.30 × 10^−22^	399.965	159	60.247	9.74 × 10^−23^	−1.12 × 10^−3^	6.28 × 10^−1^
SHBG	glaucoma	297.335	145	51.233	1.48 × 10^−12^	287.455	144	49.905	1.34 × 10^−11^	−4.96 × 10^−3^	2.77 × 10^−2^

**Table 2:** Summary of distortion results by excluding potential outliers of the bidirectional MR analysis.

Exposure	Outcome	Distortion Coefficient	*p*-Value
glaucoma	AST2ALT	−84.800	1.000
glaucoma	calcium	22.300	0.320
glaucoma	creatinine	−45.904	0.749
glaucoma	EGFR	40.933	0.755
glaucoma	LDLC	38.810	0.388
glaucoma	NAP	69.042	0.625
glaucoma	TP	47.096	0.566
BILD	glaucoma	−130.169	0.256
glucose	glaucoma	−7.264	0.799
HBA1C	glaucoma	19.595	0.691
SHBG	glaucoma	−44.425	0.796

## Data Availability

The original contributions presented in this study are included in the article/Supplementary Material. Further inquiries can be directed to the corresponding author. The data supporting the findings of this study are publicly available from several sources. The genetic data used in this study are all previously publicly accessible GWAS datasets, including FinnGen (finn-R12-H7 GLAUCOMA, accessed on 5 February 2025) and UK Biobank (https://doi.org/10.35092/yhjc.12355382, accessed on 5 February 2025). The GEO data can be accessed at NCBI (GSE216660, accessed on 12 November 2025).

## Supplementary Materials

The following supporting information can be downloaded at: https://www.mdpi.com/article/10.3390/ijms27062848/s1.

## Abbreviations

The following abbreviations are used in this manuscript:

GO	Gene Ontology
KEGG	Kyoto encyclopedia of genes and genomes
EGFR	estimated glomerular filtration rate
LDLC	LDL cholesterol
NAP	non-albumin protein
TP	total protein
AST2ALT	aspartate aminotransferase to alanine aminotransferase ratio
BILD	direct bilirubin
HBA1C	hemoglobin A1c
SHBG	sex hormone-binding globulin
IOP	intraocular pressure
RGCs	retinal ganglion cells
POAG	primary open-angle glaucoma
CKD	chronic kidney disease
GWAS	genome-wide association study
GEO	Gene Expression Omnibus
MR	Mendelian randomization
MR-PRESSO	MR pleiotropy residual sum and outlier
IVW	inverse-variance weighted
DEGs	differentially expressed genes
SNPs	single-nucleotide polymorphisms
IVs	instrumental variables
LD	linkage disequilibrium
CaMKII	Ca^2+^/Calmodulin-dependent protein kinase II
p-CaMKII	Phosphorylated Ca^2+^/Calmodulin-dependent protein kinase II
