# Supplementary material for: Unveiling Systemic Biomarkers and Metabolic Mechanisms in Glaucoma Progression from Multi-Omics Insights
Source: Int J Mol Sci. 2026 Mar 21;27(6):2848. doi: 10.3390/ijms27062848 (PMC13026555; doi:10.3390/ijms27062848)
Supplement: Supplementary file 1 [file ijms-27-02848-s001.zip › ijms-4108827-Supplementary.pdf]

Supporting Information

for

Unveiling Systemic Biomarkers and Metabolic Mechanisms in Glaucoma Progression from Multi-Omics Insights

Author: Shengshu Sun[1], Ning Xu[1], Ge Bai[1], Youhan Ao[1,2], An Wang[1], Jiaying Sun[1], Yifei Huang[1], Liqiang Wang[1,2*]

Affiliations:
[1] Medical School of Chinese People's Liberation Army, Senior Department of Ophthalmology, the Third Medical Center of Chinese PLA General Hospital, Beijing, China
[2] School of Medicine, Nankai University, Tianjin, China

*Corresponding author:
Liqiang Wang, MD, PhD (Email: liqiangw301@163.com)

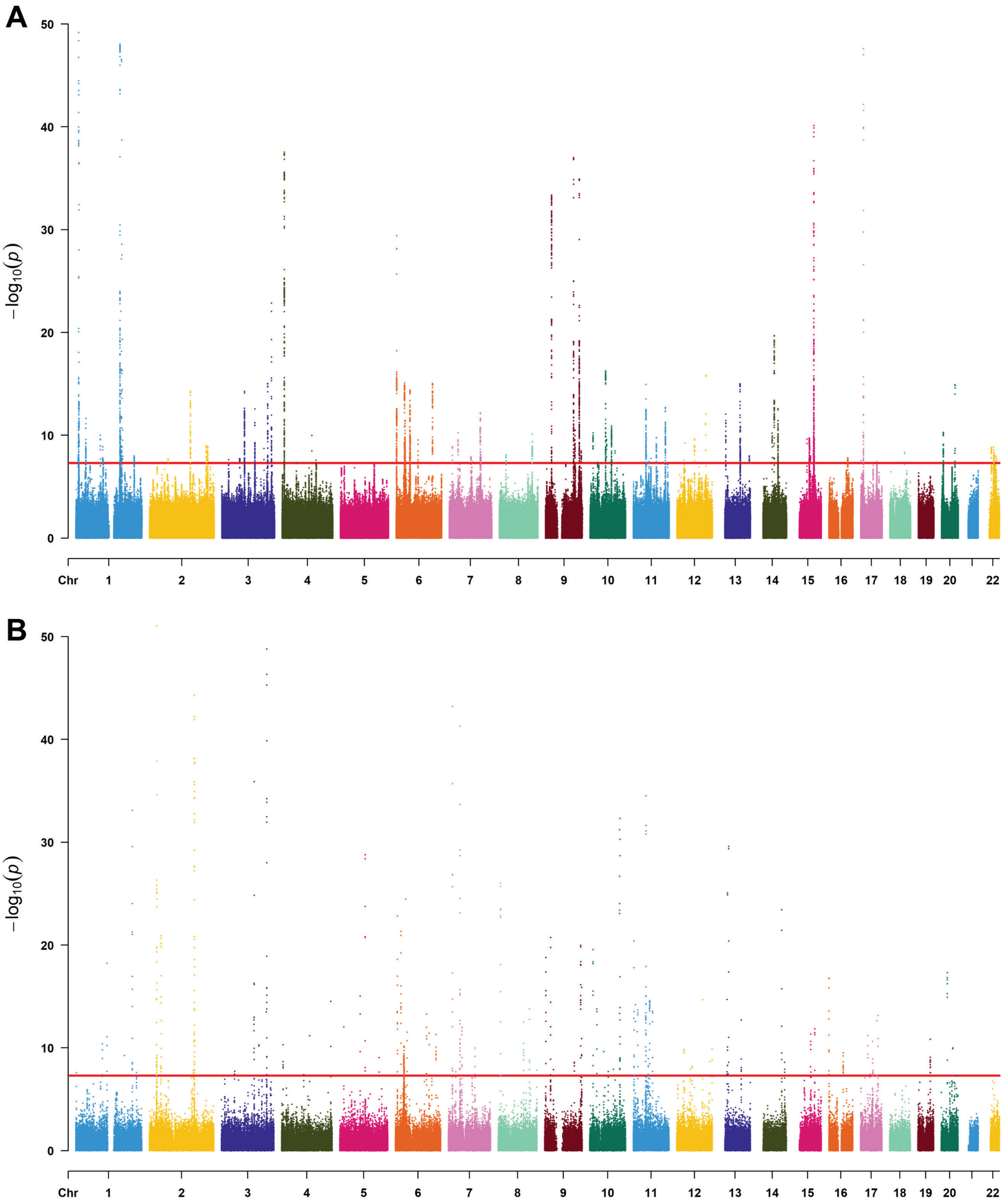

Figure S1. The Manhattan plot showed SNPs related to (A) glaucoma and (B) calcium. Red line referred to the threshold of *p*-value ($p < 5 \times 10^{-8}$).

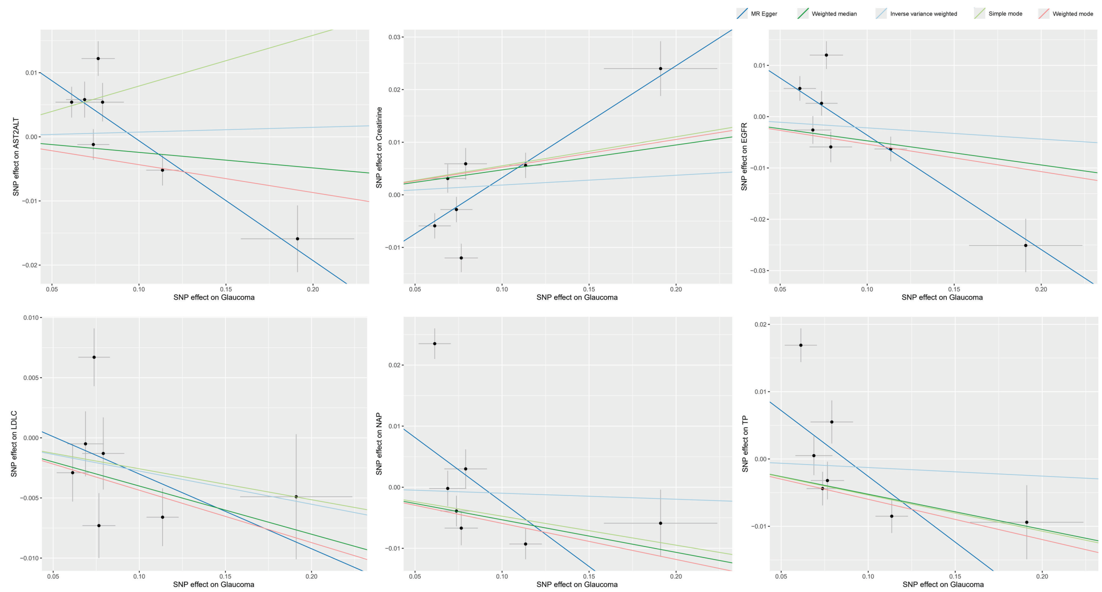

Figure S2. SNP effect of glaucoma on AST2ALT, creatinine, EGFR, LDLC, NAP and TP.

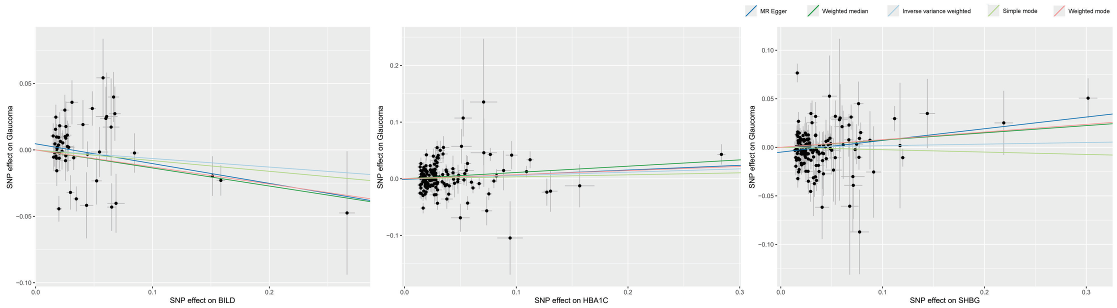

Figure S3. SNP effect of biomarkers BILD, HBA1C, and SHBG on glaucoma.

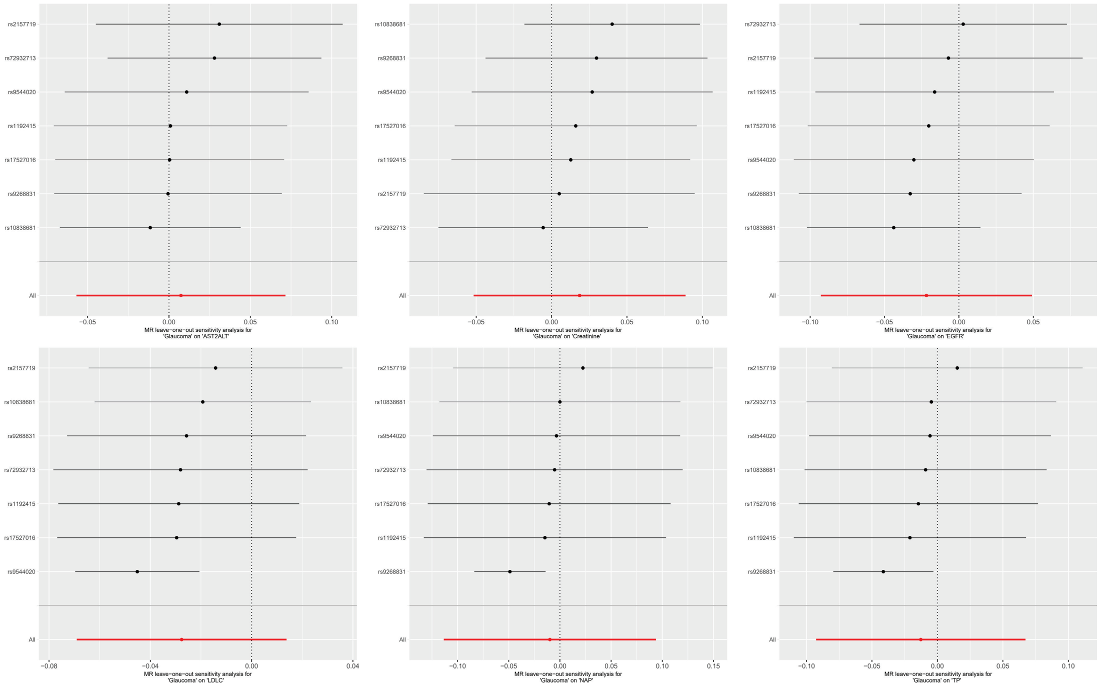

Figure S4. Leave-one-out analysis of the effect of glaucoma on biomarkers AST2ALT, creatinine, EGFR, LDLC, NAP and TP.

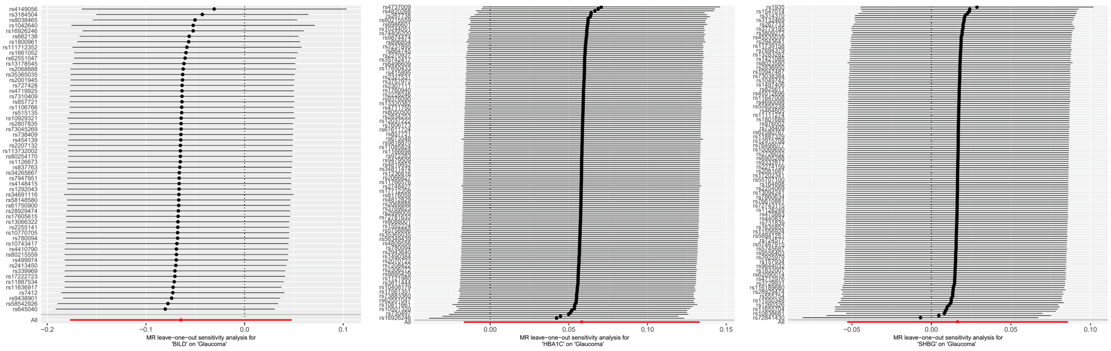

Figure S5. Leave-one-out analysis of the effect of biomarkers BILD, HBA1C, and SHBG on glaucoma.

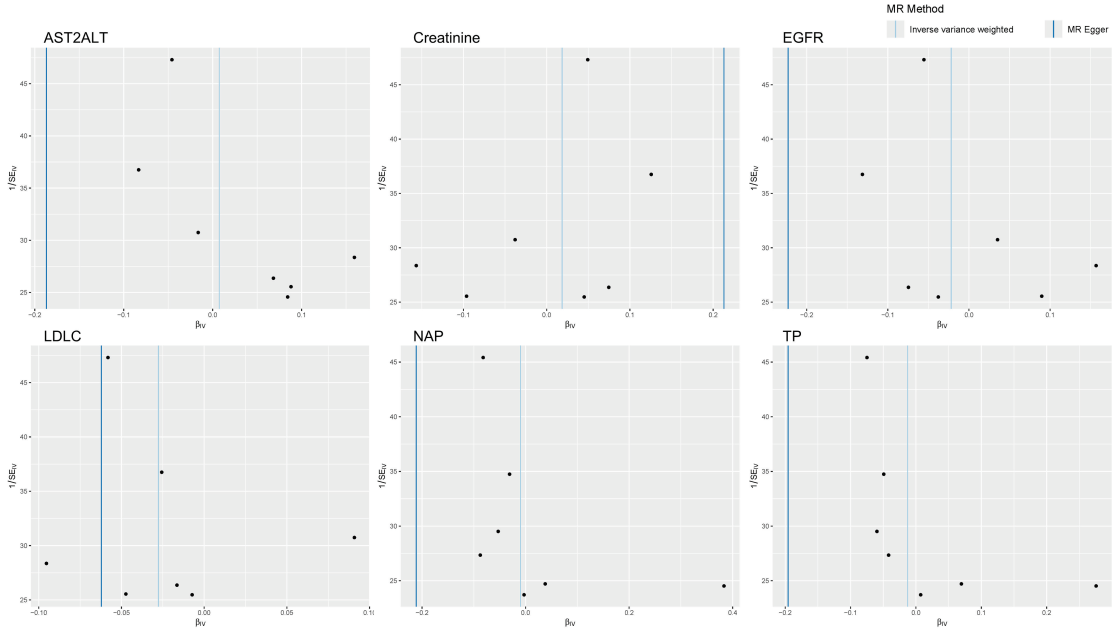

Figure S6. Funnel plots of the effect of glaucoma on biomarkers AST2ALT, creatinine, EGFR, LDLC, NAP and TP.

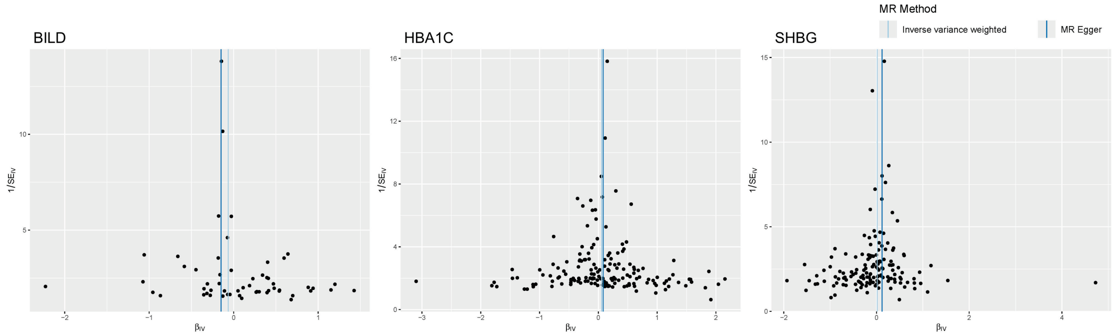

Figure S7. Funnel plots of the effect of biomarkers BILD, HBA1C, and SHBG on glaucoma.

Table S1. Summary data included in the current study.

Phenotype	Abbreviation	Trait Category	Ethnics	Number of SNPs	Number of individuals	GBE/GWAS ID
Albumin	Albumin	Liver	European	770842	291516	INI10030600
Alkaline phosphatase	ALP	Bone and Joint	European	771123	318953	INI10030610
Alanine aminotransferase	ALT	Liver	European	771124	318818	INI10030620
Apolipoprotein A	APOA	Cardiovascular	European	770832	290198	INI10030630
Apolipoprotein B	APOB	Cardiovascular	European	771110	317412	INI20030640
Aspartate aminotransferase	AST	Liver	European	771114	317763	INI10030650
AST to ALT ratio	AST2ALT	Liver	European	771114	317687	INI30030650
Direct bilirubin	BILD	Liver	European	770602	271418	INI10030660
Urea	Urea	Renal	European	771124	318724	INI10030670
Calcium	Calcium	Bone and Joint	European	770843	291843	INI10030680
Cholesterol	CHOL	Cardiovascular	European	771122	318927	INI20030690
Creatinine	Creatinine	Renal	European	771123	318800	INI10030700
C-reactive protein	CRP	Cardiovascular	European	771110	318271	INI10030710
Cystatin C	CYS	Renal	European	771123	318819	INI10030720
Estimated glomerular filtration rate	EGFR	Renal	European	771123	318800	INI30030700
Gamma glutamyl transferase	GGT	Liver	European	771123	318779	INI10030730
Glucose	Glucose	Diabetes	European	770846	291605	INI10030740
Hemoglobin A1c	HBA1C	Diabetes	European	770957	304659	INI10030750
HDL cholesterol	HDL	Cardiovascular	European	770845	291830	INI10030760
IGF-1	IGF1	Hormone	European	771103	317114	INI10030770
LDL cholesterol	LDLC	Cardiovascular	European	771119	318340	INI20030780
Lipoprotein A	LPA	Cardiovascular	European	770356	253570	INI10030790
Non-albumin protein	NAP	Renal	European	770842	291516	INI30030860
Phosphate	PHOS	Renal	European	770843	291391	INI10030810
Sex hormone-binding globulin	SHBG	Hormone	European	770809	289010	INI10030830
Total bilirubin	TBIL	Liver	European	771113	317605	INI10030840
Testosterone	TES	Hormone	European	770814	289117	INI10030850
Total protein	TP	Renal	European	770842	291516	INI10030860
Triglycerides	TRIG	Cardiovascular	European	771121	318674	INI10030870
Urate	Urate	Renal	European	771120	318526	INI10030880
Creatinine in urine	UCR	Renal	European	771041	310240	INI10030510
Potassium in urine	URK	Renal	European	771037	309559	INI10030520
Microalbumin in urine	URMA	Renal	European	765442	95811	INI10030500
Sodium in urine	URNA	Renal	European	771030	309585	INI10030530
Vitamin D	VITD	Bone and Joint	European	770977	304818	INI10030890
Glaucoma	Glaucoma	—	European	16380466	218792	finn-R12-H7

Table S2. Forward MR analysis results of the effect of glaucoma on 35 blood and urine biomarkers.

Exposure	Outcome	MR Analysis method	SNPs	β	SE	*p*-value	*p*-adjust	lo_ci	up_ci	OR	95% CI_low	95% CI_high
glaucoma	Albumin	MR Egger	7	−0.026	0.057	0.662	0.773	−0.137	0.085	0.974	0.872	1.088
		Weighted median	7	−0.012	0.016	0.444	0.897	−0.044	0.019	0.988	0.957	1.019
		Inverse variance weighted	7	−0.005	0.016	0.768	0.822	−0.037	0.027	0.995	0.964	1.028
		Simple mode	7	−0.017	0.026	0.529	0.962	−0.067	0.033	0.983	0.935	1.034
		Weighted mode	7	−0.017	0.020	0.432	0.954	−0.055	0.022	0.984	0.946	1.022
glaucoma	ALP	MR Egger	7	0.020	0.095	0.842	0.842	−0.167	0.207	1.020	0.846	1.230
		Weighted median	7	0.010	0.016	0.542	0.542	−0.022	0.042	1.010	0.978	1.043
		Inverse variance weighted	7	0.027	0.027	0.320	0.480	−0.026	0.081	1.028	0.974	1.084
		Simple mode	7	−0.004	0.021	0.870	0.870	−0.045	0.038	0.996	0.956	1.039
		Weighted mode	7	0.012	0.019	0.552	0.552	−0.025	0.050	1.012	0.975	1.051
glaucoma	ALT	MR Egger	7	0.075	0.061	0.273	0.690	−0.045	0.195	1.078	0.956	1.215
		Weighted median	7	0.010	0.017	0.550	0.897	−0.023	0.044	1.010	0.977	1.044
		Inverse variance weighted	7	0.009	0.020	0.666	0.822	−0.030	0.047	1.009	0.970	1.048
		Simple mode	7	0.012	0.031	0.712	0.962	−0.048	0.072	1.012	0.953	1.075
		Weighted mode	7	0.012	0.023	0.617	0.954	−0.032	0.056	1.012	0.968	1.058
glaucoma	APOA	MR Egger	7	−0.206	0.211	0.375	0.745	−0.619	0.208	0.814	0.538	1.231
		Weighted median	7	0.013	0.018	0.477	0.536	−0.023	0.049	1.013	0.977	1.051
		Inverse variance weighted	7	0.082	0.072	0.255	0.660	−0.059	0.222	1.085	0.943	1.249
		Simple mode	7	−0.005	0.020	0.792	0.792	−0.044	0.033	0.995	0.957	1.034
		Weighted mode	7	0.000	0.016	0.991	0.991	−0.032	0.032	1.000	0.969	1.032
glaucoma	APOB	MR Egger	7	−0.007	0.094	0.947	0.947	−0.190	0.177	0.993	0.827	1.194
		Weighted median	7	−0.024	0.016	0.132	0.359	−0.055	0.007	0.976	0.946	1.007
		Inverse variance weighted	7	−0.026	0.027	0.330	0.660	−0.079	0.027	0.974	0.924	1.027
		Simple mode	7	−0.024	0.020	0.266	0.569	−0.063	0.015	0.976	0.939	1.015
		Weighted mode	7	−0.029	0.016	0.134	0.356	−0.061	0.004	0.972	0.941	1.004
glaucoma	AST	MR Egger	7	−0.115	0.098	0.296	0.690	−0.308	0.078	0.891	0.735	1.081
		Weighted median	7	0.006	0.020	0.769	0.897	−0.033	0.044	1.006	0.968	1.045
		Inverse variance weighted	7	0.025	0.034	0.467	0.822	−0.042	0.091	1.025	0.959	1.096
		Simple mode	7	0.001	0.025	0.962	0.962	−0.049	0.051	1.001	0.953	1.052

		Weighted mode	7	−0.015	0.019	0.463	0.954	−0.051	0.022	0.986	0.950	1.022
		MR Egger	7	−0.187	0.068	0.040	0.280	−0.320	−0.054	0.830	0.726	0.947
		Weighted median	7	−0.024	0.020	0.215	0.897	−0.063	0.014	0.976	0.939	1.014
glaucoma	AST2ALT	Inverse variance weighted	7	0.007	0.033	0.822	0.822	−0.057	0.072	1.007	0.945	1.074
		Simple mode	7	0.079	0.067	0.283	0.962	−0.052	0.210	1.082	0.949	1.234
		Weighted mode	7	−0.043	0.019	0.065	0.456	−0.081	−0.006	0.957	0.922	0.994
		MR Egger	7	0.040	0.072	0.607	0.773	−0.102	0.182	1.041	0.903	1.199
		Weighted median	7	0.006	0.019	0.740	0.897	−0.031	0.044	1.006	0.969	1.045
glaucoma	BILD	Inverse variance weighted	7	0.025	0.021	0.223	0.791	−0.015	0.066	1.026	0.985	1.068
		Simple mode	7	−0.008	0.032	0.821	0.962	−0.071	0.055	0.992	0.932	1.057
		Weighted mode	7	−0.002	0.039	0.954	0.954	−0.078	0.073	0.998	0.925	1.076
		MR Egger	7	0.051	0.102	0.634	0.691	−0.148	0.250	1.053	0.863	1.285
		Weighted median	7	0.001	0.016	0.944	0.944	−0.030	0.032	1.001	0.971	1.032
glaucoma	Urea	Inverse variance weighted	7	−0.019	0.031	0.541	0.975	−0.079	0.041	0.981	0.924	1.042
		Simple mode	7	0.014	0.020	0.499	0.734	−0.025	0.053	1.014	0.976	1.055
		Weighted mode	7	0.005	0.019	0.799	0.861	−0.033	0.043	1.005	0.968	1.044
		MR Egger	7	0.025	0.072	0.742	0.842	−0.117	0.167	1.026	0.890	1.182
		Weighted median	7	0.056	0.020	0.005	0.015	0.017	0.095	1.058	1.017	1.100
glaucoma	CA	Inverse variance weighted	7	0.043	0.021	0.039	0.118	0.002	0.084	1.044	1.002	1.088
		Simple mode	7	0.064	0.035	0.117	0.351	−0.005	0.133	1.066	0.995	1.142
		Weighted mode	7	0.078	0.030	0.040	0.119	0.020	0.137	1.082	1.020	1.147
		MR Egger	7	−0.109	0.053	0.092	0.736	−0.212	−0.006	0.896	0.809	0.994
		Weighted median	7	−0.027	0.019	0.151	0.359	−0.063	0.010	0.974	0.939	1.010
glaucoma	CHOL	Inverse variance weighted	7	−0.002	0.021	0.926	0.953	−0.043	0.039	0.998	0.958	1.040
		Simple mode	7	−0.025	0.044	0.595	0.680	−0.112	0.062	0.975	0.894	1.064
		Weighted mode	7	−0.037	0.019	0.097	0.356	−0.075	0.000	0.963	0.928	1.000
		MR Egger	7	0.213	0.085	0.054	0.323	0.047	0.379	1.237	1.048	1.461
		Weighted median	7	0.047	0.019	0.014	0.049	0.010	0.085	1.049	1.010	1.089
glaucoma	creatinine	Inverse variance weighted	7	0.019	0.036	0.604	0.975	−0.052	0.089	1.019	0.950	1.093
		Simple mode	7	0.055	0.028	0.095	0.372	0.001	0.110	1.057	1.001	1.116
		Weighted mode	7	0.053	0.023	0.058	0.174	0.008	0.097	1.054	1.009	1.102
glaucoma	CRP	MR Egger	7	−0.032	0.226	0.891	0.947	−0.475	0.410	0.968	0.622	1.507

		Weighted median	7	−0.012	0.019	0.536	0.536	−0.050	0.026	0.988	0.951	1.026
		Inverse variance weighted	7	0.004	0.065	0.953	0.953	−0.123	0.131	1.004	0.884	1.140
		Simple mode	7	0.013	0.023	0.583	0.680	−0.032	0.059	1.013	0.969	1.060
		Weighted mode	7	−0.008	0.019	0.682	0.909	−0.046	0.029	0.992	0.955	1.030
		MR Egger	7	0.182	0.122	0.194	0.352	−0.056	0.420	1.200	0.945	1.523
		Weighted median	7	−0.010	0.018	0.562	0.749	−0.046	0.025	0.990	0.955	1.025
glaucoma	CYS	Inverse variance weighted	7	−0.046	0.047	0.327	0.975	−0.137	0.046	0.955	0.872	1.047
		Simple mode	7	0.009	0.021	0.699	0.776	−0.033	0.051	1.009	0.967	1.052
		Weighted mode	7	−0.007	0.018	0.704	0.845	−0.042	0.028	0.993	0.959	1.028
		MR Egger	7	−0.223	0.083	0.044	0.323	−0.386	−0.060	0.800	0.680	0.942
		Weighted median	7	−0.047	0.020	0.016	0.049	−0.085	−0.009	0.954	0.918	0.991
glaucoma	EGFR	Inverse variance weighted	7	−0.022	0.036	0.547	0.975	−0.093	0.049	0.978	0.911	1.050
		Simple mode	7	−0.054	0.030	0.124	0.372	−0.112	0.005	0.948	0.894	1.005
		Weighted mode	7	−0.054	0.022	0.053	0.174	−0.097	−0.010	0.948	0.907	0.990
		MR Egger	7	0.024	0.043	0.608	0.773	−0.061	0.109	1.024	0.940	1.115
		Weighted median	7	0.006	0.015	0.706	0.897	−0.024	0.036	1.006	0.976	1.037
glaucoma	GGT	Inverse variance weighted	7	0.015	0.013	0.226	0.791	−0.009	0.040	1.015	0.991	1.040
		Simple mode	7	−0.007	0.027	0.801	0.962	−0.061	0.046	0.993	0.941	1.048
		Weighted mode	7	−0.004	0.021	0.851	0.954	−0.045	0.036	0.996	0.956	1.037
		MR Egger	7	0.105	0.125	0.437	0.874	−0.139	0.349	1.111	0.870	1.418
		Weighted median	7	0.018	0.022	0.407	0.521	−0.024	0.060	1.018	0.976	1.062
glaucoma	GLU	Inverse variance weighted	7	0.059	0.036	0.103	0.207	−0.012	0.130	1.061	0.988	1.139
		Simple mode	7	0.001	0.024	0.964	0.964	−0.047	0.049	1.001	0.954	1.050
		Weighted mode	7	0.004	0.034	0.917	0.917	−0.063	0.071	1.004	0.939	1.073
		MR Egger	7	0.015	0.125	0.909	0.909	−0.231	0.261	1.015	0.794	1.298
		Weighted median	7	0.014	0.021	0.521	0.521	−0.028	0.056	1.014	0.972	1.057
glaucoma	HBA1C	Inverse variance weighted	7	0.039	0.036	0.283	0.283	−0.032	0.110	1.040	0.968	1.116
		Simple mode	7	−0.033	0.027	0.270	0.540	−0.086	0.020	0.968	0.918	1.020
		Weighted mode	7	−0.031	0.052	0.578	0.917	−0.133	0.072	0.970	0.876	1.074
		MR Egger	7	−0.215	0.272	0.465	0.745	−0.749	0.319	0.807	0.473	1.375
glaucoma	HDL	Weighted median	7	−0.017	0.018	0.342	0.536	−0.052	0.018	0.983	0.949	1.018
		Inverse variance weighted	7	0.071	0.087	0.412	0.660	−0.099	0.242	1.074	0.906	1.273

		Simple mode	7	−0.024	0.018	0.233	0.569	−0.059	0.011	0.976	0.942	1.012
		Weighted mode	7	−0.024	0.016	0.183	0.361	−0.055	0.007	0.976	0.946	1.007
glaucoma	IGF1	MR Egger	7	−0.055	0.207	0.801	0.964	−0.461	0.351	0.946	0.631	1.420
		Weighted median	7	−0.015	0.015	0.311	0.543	−0.045	0.014	0.985	0.956	1.014
		Inverse variance weighted	7	0.027	0.061	0.655	0.906	−0.092	0.146	1.027	0.912	1.157
		Simple mode	7	−0.016	0.020	0.466	0.699	−0.055	0.024	0.985	0.947	1.024
		Weighted mode	7	−0.017	0.015	0.297	0.785	−0.046	0.012	0.983	0.955	1.012
glaucoma	LDLC	MR Egger	7	−0.062	0.072	0.426	0.745	−0.203	0.079	0.940	0.816	1.082
		Weighted median	7	−0.040	0.015	0.009	0.073	−0.070	−0.010	0.961	0.932	0.990
		Inverse variance weighted	7	−0.028	0.021	0.191	0.660	−0.069	0.014	0.973	0.933	1.014
		Simple mode	7	−0.026	0.022	0.284	0.569	−0.069	0.017	0.975	0.934	1.017
		Weighted mode	7	−0.044	0.019	0.066	0.356	−0.082	−0.006	0.957	0.922	0.994
glaucoma	LPA	MR Egger	7	−0.059	0.042	0.217	0.745	−0.140	0.023	0.943	0.869	1.023
		Weighted median	7	−0.023	0.017	0.180	0.359	−0.056	0.011	0.977	0.945	1.011
		Inverse variance weighted	7	−0.015	0.013	0.257	0.660	−0.041	0.011	0.985	0.960	1.011
		Simple mode	7	−0.022	0.025	0.400	0.639	−0.070	0.026	0.978	0.932	1.026
		Weighted mode	7	−0.026	0.019	0.226	0.361	−0.064	0.012	0.974	0.938	1.012
glaucoma	NAP	MR Egger	7	−0.211	0.159	0.240	0.361	−0.522	0.100	0.810	0.593	1.105
		Weighted median	7	−0.053	0.019	0.004	0.025	−0.090	−0.017	0.948	0.914	0.983
		Inverse variance weighted	7	−0.010	0.053	0.852	0.975	−0.113	0.094	0.990	0.893	1.098
		Simple mode	7	−0.047	0.021	0.065	0.372	−0.089	−0.006	0.954	0.915	0.994
		Weighted mode	7	−0.059	0.018	0.017	0.102	−0.095	−0.024	0.943	0.910	0.977
glaucoma	PHOS	MR Egger	7	−0.108	0.052	0.090	0.323	−0.210	−0.007	0.897	0.811	0.993
		Weighted median	7	−0.020	0.019	0.289	0.496	−0.057	0.017	0.980	0.945	1.017
		Inverse variance weighted	7	0.001	0.021	0.975	0.975	−0.040	0.042	1.001	0.961	1.042
		Simple mode	7	0.055	0.044	0.257	0.513	−0.031	0.140	1.056	0.970	1.150
		Weighted mode	7	−0.033	0.025	0.222	0.444	−0.081	0.015	0.967	0.922	1.015
glaucoma	SHBG	MR Egger	7	−0.007	0.137	0.964	0.964	−0.275	0.262	0.993	0.759	1.300
		Weighted median	7	−0.007	0.017	0.707	0.707	−0.041	0.028	0.993	0.960	1.028
		Inverse variance weighted	7	−0.005	0.039	0.906	0.906	−0.082	0.072	0.995	0.922	1.075
		Simple mode	7	−0.005	0.022	0.825	0.825	−0.047	0.037	0.995	0.954	1.038
		Weighted mode	7	0.002	0.019	0.935	0.935	−0.036	0.039	1.002	0.965	1.040

		MR Egger	7	−0.017	0.069	0.814	0.814	−0.153	0.118	0.983	0.858	1.126
		Weighted median	7	0.002	0.015	0.908	0.908	−0.028	0.031	1.002	0.972	1.032
glaucoma	TBIL	Inverse variance weighted	7	−0.005	0.020	0.784	0.822	−0.045	0.034	0.995	0.956	1.034
		Simple mode	7	−0.002	0.021	0.934	0.962	−0.042	0.039	0.998	0.958	1.040
		Weighted mode	7	0.001	0.018	0.953	0.954	−0.034	0.036	1.001	0.966	1.037
		MR Egger	7	0.059	0.041	0.212	0.636	−0.022	0.140	1.061	0.978	1.150
		Weighted median	7	0.015	0.016	0.362	0.543	−0.017	0.047	1.015	0.983	1.048
glaucoma	TES	Inverse variance weighted	7	0.009	0.014	0.495	0.906	−0.017	0.036	1.009	0.983	1.037
		Simple mode	7	0.028	0.025	0.312	0.699	−0.021	0.076	1.028	0.979	1.079
		Weighted mode	7	0.014	0.021	0.523	0.785	−0.026	0.054	1.014	0.974	1.056
		MR Egger	7	−0.195	0.114	0.146	0.350	−0.418	0.027	0.822	0.658	1.028
		Weighted median	7	−0.052	0.017	0.002	0.025	−0.086	−0.019	0.949	0.918	0.981
glaucoma	TP	Inverse variance weighted	7	−0.013	0.041	0.755	0.975	−0.093	0.067	0.987	0.912	1.070
		Simple mode	7	−0.054	0.021	0.044	0.372	−0.095	−0.012	0.948	0.909	0.988
		Weighted mode	7	−0.060	0.016	0.008	0.102	−0.090	−0.029	0.942	0.914	0.971
		MR Egger	7	0.074	0.173	0.686	0.914	−0.265	0.413	1.077	0.767	1.512
		Weighted median	7	0.015	0.018	0.404	0.536	−0.021	0.051	1.015	0.980	1.053
glaucoma	TRIG	Inverse variance weighted	7	−0.011	0.051	0.836	0.953	−0.110	0.089	0.990	0.895	1.093
		Simple mode	7	0.032	0.027	0.279	0.569	−0.021	0.084	1.032	0.980	1.088
		Weighted mode	7	0.003	0.020	0.889	0.991	−0.036	0.042	1.003	0.964	1.043
		MR Egger	7	0.216	0.111	0.108	0.323	0.000	0.433	1.242	1.000	1.542
		Weighted median	7	−0.026	0.017	0.139	0.305	−0.060	0.008	0.975	0.942	1.008
glaucoma	Urate	Inverse variance weighted	7	−0.004	0.044	0.923	0.975	−0.090	0.081	0.996	0.914	1.085
		Simple mode	7	−0.049	0.037	0.233	0.513	−0.121	0.023	0.952	0.886	1.024
		Weighted mode	7	−0.032	0.022	0.184	0.442	−0.075	0.010	0.968	0.928	1.010
		MR Egger	7	0.109	0.099	0.323	0.431	−0.086	0.303	1.115	0.918	1.354
		Weighted median	7	−0.010	0.017	0.547	0.749	−0.043	0.023	0.990	0.958	1.023
glaucoma	UCR	Inverse variance weighted	7	0.039	0.030	0.192	0.975	−0.020	0.098	1.040	0.980	1.103
		Simple mode	7	−0.011	0.018	0.550	0.734	−0.046	0.023	0.989	0.955	1.024
		Weighted mode	7	−0.012	0.016	0.501	0.667	−0.043	0.020	0.988	0.957	1.020
glaucoma	URK	MR Egger	7	0.106	0.073	0.205	0.352	−0.037	0.249	1.112	0.964	1.282
		Weighted median	7	−0.006	0.018	0.718	0.783	−0.041	0.028	0.994	0.960	1.029

		Inverse variance weighted	7	0.023	0.024	0.342	0.975	−0.024	0.069	1.023	0.976	1.072
		Simple mode	7	0.003	0.028	0.908	0.908	−0.051	0.058	1.003	0.950	1.060
		Weighted mode	7	−0.004	0.021	0.861	0.861	−0.045	0.037	0.996	0.956	1.038
		MR Egger	7	0.051	0.095	0.611	0.691	−0.135	0.237	1.053	0.874	1.268
		Weighted median	7	0.041	0.029	0.153	0.305	−0.015	0.098	1.042	0.985	1.103
glaucoma	URMA	Inverse variance weighted	7	0.021	0.027	0.446	0.975	−0.033	0.075	1.021	0.968	1.078
		Simple mode	7	0.018	0.047	0.711	0.776	−0.074	0.110	1.018	0.929	1.116
		Weighted mode	7	0.037	0.035	0.335	0.574	−0.032	0.106	1.037	0.969	1.111
		MR Egger	7	−0.007	0.049	0.887	0.887	−0.102	0.088	0.993	0.903	1.092
		Weighted median	7	0.006	0.016	0.706	0.783	−0.025	0.037	1.006	0.975	1.038
glaucoma	URNA	Inverse variance weighted	7	0.006	0.014	0.687	0.975	−0.022	0.033	1.006	0.978	1.034
		Simple mode	7	0.033	0.032	0.347	0.595	−0.030	0.096	1.033	0.970	1.100
		Weighted mode	7	−0.024	0.031	0.462	0.667	−0.086	0.037	0.976	0.918	1.037
		MR Egger	7	−0.091	0.046	0.106	0.317	−0.182	−0.001	0.913	0.834	0.999
		Weighted median	7	−0.028	0.016	0.090	0.135	−0.060	0.004	0.973	0.942	1.004
glaucoma	VITD	Inverse variance weighted	7	−0.006	0.017	0.725	0.725	−0.040	0.028	0.994	0.960	1.028
		Simple mode	7	−0.016	0.021	0.471	0.707	−0.058	0.025	0.984	0.943	1.026
		Weighted mode	7	−0.027	0.018	0.196	0.294	−0.062	0.009	0.974	0.939	1.009

Table S3. Backward MR analysis results of the effect of 35 blood and urine biomarkers on glaucoma.

Exposure	Outcome	MR Analysis method	SNPs	β	SE	*p*-value	*p*-adjust	lo_ci	up_ci	OR	95% CI_low	95% CI_high
Albumin	glaucoma	MR Egger	113	−0.020	0.096	0.832	0.943	−0.208	0.167	0.980	0.812	1.182
		Weighted median	113	−0.010	0.054	0.859	0.860	−0.115	0.096	0.990	0.892	1.100
		Inverse variance weighted	113	0.087	0.052	0.094	0.329	−0.015	0.188	1.090	0.985	1.207
		Simple mode	113	−0.009	0.106	0.933	0.933	−0.216	0.198	0.991	0.805	1.219
		Weighted mode	113	−0.020	0.060	0.745	0.745	−0.138	0.098	0.981	0.871	1.103
ALP	glaucoma	MR Egger	148	−0.082	0.056	0.145	0.428	−0.192	0.028	0.921	0.825	1.028
		Weighted median	148	0.030	0.039	0.438	0.656	−0.046	0.106	1.031	0.955	1.112
		Inverse variance weighted	148	−0.032	0.034	0.336	0.922	−0.098	0.033	0.968	0.907	1.034
		Simple mode	148	0.044	0.079	0.578	0.750	−0.111	0.200	1.045	0.895	1.221
		Weighted mode	148	0.003	0.038	0.935	0.935	−0.071	0.077	1.003	0.932	1.080
ALT	glaucoma	MR Egger	112	0.008	0.109	0.943	0.943	−0.205	0.221	1.008	0.815	1.247
		Weighted median	112	−0.014	0.073	0.848	0.860	−0.156	0.128	0.986	0.855	1.137
		Inverse variance weighted	112	0.008	0.054	0.885	0.984	−0.097	0.113	1.008	0.907	1.120
		Simple mode	112	−0.124	0.127	0.330	0.933	−0.373	0.125	0.883	0.689	1.133
		Weighted mode	112	−0.039	0.082	0.632	0.745	−0.200	0.121	0.961	0.819	1.129
APOA	glaucoma	MR Egger	126	0.047	0.060	0.436	0.909	−0.071	0.164	1.048	0.932	1.179
		Weighted median	126	−0.002	0.037	0.957	0.957	−0.075	0.071	0.998	0.928	1.073
		Inverse variance weighted	126	0.037	0.039	0.345	0.590	−0.039	0.112	1.037	0.961	1.119
		Simple mode	126	0.051	0.068	0.452	0.969	−0.082	0.185	1.053	0.921	1.203
		Weighted mode	126	0.015	0.035	0.675	0.915	−0.055	0.084	1.015	0.947	1.088
APOB	glaucoma	MR Egger	145	−0.033	0.041	0.418	0.909	−0.113	0.047	0.967	0.893	1.048
		Weighted median	145	−0.029	0.033	0.385	0.681	−0.093	0.036	0.972	0.911	1.037
		Inverse variance weighted	145	−0.045	0.029	0.124	0.495	−0.102	0.012	0.956	0.903	1.012
		Simple mode	145	−0.056	0.064	0.382	0.969	−0.182	0.070	0.945	0.833	1.072
		Weighted mode	145	−0.027	0.029	0.350	0.915	−0.084	0.030	0.973	0.920	1.030
AST	glaucoma	MR Egger	124	0.022	0.097	0.821	0.943	−0.168	0.212	1.022	0.845	1.237
		Weighted median	124	−0.012	0.065	0.860	0.860	−0.139	0.116	0.989	0.870	1.123
		Inverse variance weighted	124	−0.001	0.051	0.984	0.984	−0.101	0.099	0.999	0.904	1.104
		Simple mode	124	−0.061	0.136	0.654	0.933	−0.329	0.206	0.941	0.720	1.229

		Weighted mode	124	−0.035	0.078	0.651	0.745	−0.188	0.117	0.965	0.829	1.124
AST2ALT	glaucoma	MR Egger	101	−0.033	0.111	0.768	0.943	−0.250	0.185	0.968	0.779	1.203
		Weighted median	101	0.134	0.073	0.067	0.234	−0.009	0.277	1.143	0.991	1.320
		Inverse variance weighted	101	0.099	0.059	0.093	0.329	−0.017	0.214	1.104	0.984	1.239
		Simple mode	101	0.020	0.151	0.893	0.933	−0.275	0.315	1.020	0.760	1.371
		Weighted mode	101	0.117	0.073	0.113	0.397	−0.027	0.261	1.124	0.974	1.298
BILD	glaucoma	MR Egger	56	−0.149	0.084	0.084	0.585	−0.314	0.017	0.862	0.730	1.017
		Weighted median	56	−0.136	0.059	0.022	0.152	−0.252	−0.020	0.873	0.777	0.980
		Inverse variance weighted	56	−0.065	0.057	0.260	0.454	−0.177	0.048	0.937	0.838	1.049
		Simple mode	56	−0.080	0.155	0.607	0.933	−0.385	0.224	0.923	0.680	1.251
		Weighted mode	56	−0.128	0.053	0.019	0.132	−0.232	−0.024	0.880	0.793	0.976
Urea	glaucoma	MR Egger	89	−0.037	0.146	0.799	0.945	−0.324	0.249	0.963	0.723	1.283
		Weighted median	89	−0.076	0.072	0.290	0.701	−0.216	0.065	0.927	0.806	1.067
		Inverse variance weighted	89	−0.098	0.069	0.156	0.572	−0.234	0.037	0.907	0.792	1.038
		Simple mode	89	0.004	0.137	0.974	0.991	−0.265	0.274	1.004	0.767	1.315
		Weighted mode	89	−0.094	0.078	0.232	0.849	−0.247	0.059	0.910	0.781	1.061
calcium	glaucoma	MR Egger	78	0.103	0.095	0.286	0.428	−0.084	0.290	1.108	0.919	1.336
		Weighted median	78	0.005	0.065	0.944	0.944	−0.122	0.132	1.005	0.885	1.141
		Inverse variance weighted	78	−0.006	0.057	0.922	0.922	−0.116	0.105	0.994	0.890	1.111
		Simple mode	78	−0.033	0.105	0.750	0.750	−0.239	0.172	0.967	0.787	1.188
		Weighted mode	78	−0.005	0.054	0.920	0.935	−0.112	0.101	0.995	0.894	1.106
CHOL	glaucoma	MR Egger	149	−0.077	0.039	0.050	0.400	−0.152	−0.001	0.926	0.859	0.999
		Weighted median	149	−0.023	0.033	0.489	0.681	−0.087	0.041	0.978	0.917	1.042
		Inverse variance weighted	149	−0.019	0.026	0.475	0.590	−0.070	0.033	0.982	0.932	1.033
		Simple mode	149	−0.002	0.060	0.969	0.969	−0.120	0.115	0.998	0.887	1.122
		Weighted mode	149	−0.024	0.030	0.434	0.915	−0.083	0.036	0.976	0.920	1.036
creatinine	glaucoma	MR Egger	136	0.058	0.092	0.530	0.945	−0.122	0.237	1.059	0.885	1.267
		Weighted median	136	−0.051	0.051	0.319	0.701	−0.152	0.050	0.950	0.859	1.051
		Inverse variance weighted	136	−0.009	0.041	0.829	0.985	−0.090	0.072	0.991	0.914	1.075
		Simple mode	136	0.150	0.131	0.255	0.935	−0.107	0.407	1.162	0.898	1.502
		Weighted mode	136	−0.095	0.069	0.174	0.849	−0.231	0.041	0.910	0.794	1.042
CRP	glaucoma	MR Egger	94	−0.006	0.073	0.935	0.935	−0.150	0.138	0.994	0.861	1.148

		Weighted median	94	0.043	0.051	0.401	0.681	−0.057	0.143	1.044	0.944	1.154
		Inverse variance weighted	94	0.013	0.050	0.798	0.798	−0.085	0.110	1.013	0.919	1.116
		Simple mode	94	0.014	0.117	0.903	0.969	−0.215	0.244	1.014	0.806	1.276
		Weighted mode	94	0.001	0.039	0.982	0.982	−0.075	0.076	1.001	0.928	1.079
CYS	glaucoma	MR Egger	124	−0.014	0.106	0.896	0.945	−0.221	0.194	0.986	0.801	1.214
		Weighted median	124	0.006	0.057	0.921	0.921	−0.106	0.118	1.006	0.899	1.125
		Inverse variance weighted	124	−0.088	0.055	0.105	0.572	−0.195	0.018	0.915	0.823	1.019
		Simple mode	124	−0.217	0.134	0.108	0.916	−0.481	0.046	0.805	0.618	1.047
		Weighted mode	124	0.003	0.069	0.963	0.963	−0.133	0.139	1.003	0.876	1.149
EGFR	glaucoma	MR Egger	137	−0.065	0.095	0.494	0.945	−0.252	0.122	0.937	0.777	1.129
		Weighted median	137	0.056	0.054	0.293	0.701	−0.049	0.162	1.058	0.952	1.176
		Inverse variance weighted	137	0.008	0.042	0.858	0.985	−0.075	0.090	1.008	0.927	1.095
		Simple mode	137	−0.199	0.143	0.167	0.916	−0.479	0.081	0.820	0.620	1.085
		Weighted mode	137	0.093	0.078	0.235	0.849	−0.060	0.246	1.097	0.942	1.279
GGT	glaucoma	MR Egger	129	−0.033	0.064	0.607	0.943	−0.158	0.092	0.968	0.854	1.097
		Weighted median	129	−0.040	0.043	0.349	0.611	−0.125	0.044	0.961	0.883	1.045
		Inverse variance weighted	129	−0.047	0.036	0.184	0.429	−0.117	0.022	0.954	0.889	1.023
		Simple mode	129	−0.027	0.092	0.770	0.933	−0.207	0.153	0.973	0.813	1.166
		Weighted mode	129	−0.016	0.041	0.699	0.745	−0.096	0.064	0.984	0.909	1.066
GLU	glaucoma	MR Egger	68	0.193	0.130	0.142	0.195	−0.061	0.448	1.213	0.940	1.565
		Weighted median	68	0.281	0.075	0.0002	0.0004	0.134	0.427	1.324	1.143	1.533
		Inverse variance weighted	68	0.186	0.068	0.007	0.013	0.052	0.320	1.204	1.053	1.377
		Simple mode	68	0.196	0.154	0.207	0.413	−0.105	0.497	1.217	0.900	1.645
		Weighted mode	68	0.267	0.084	0.002	0.005	0.102	0.432	1.306	1.107	1.540
HBA1C	glaucoma	MR Egger	161	0.083	0.064	0.195	0.195	−0.042	0.208	1.086	0.959	1.231
		Weighted median	161	0.111	0.045	0.014	0.014	0.023	0.199	1.117	1.023	1.221
		Inverse variance weighted	161	0.058	0.038	0.128	0.128	−0.017	0.133	1.060	0.983	1.142
		Simple mode	161	0.035	0.099	0.726	0.726	−0.160	0.230	1.036	0.852	1.258
		Weighted mode	161	0.072	0.042	0.087	0.087	−0.010	0.155	1.075	0.990	1.168
HDL	glaucoma	MR Egger	140	0.021	0.057	0.717	0.935	−0.091	0.132	1.021	0.913	1.141
		Weighted median	140	−0.024	0.039	0.535	0.681	−0.099	0.052	0.976	0.905	1.053
		Inverse variance weighted	140	0.037	0.036	0.304	0.590	−0.033	0.107	1.038	0.967	1.113

		Simple mode	140	0.068	0.067	0.310	0.969	−0.063	0.200	1.071	0.939	1.221
		Weighted mode	140	−0.001	0.032	0.967	0.982	−0.065	0.062	0.999	0.937	1.064
		MR Egger	137	−0.092	0.101	0.363	0.545	−0.289	0.105	0.912	0.749	1.111
		Weighted median	137	0.037	0.049	0.454	0.454	−0.060	0.134	1.038	0.942	1.143
IGF1	glaucoma	Inverse variance weighted	137	0.027	0.050	0.596	0.642	−0.072	0.126	1.027	0.930	1.134
		Simple mode	137	0.021	0.105	0.843	0.843	−0.185	0.227	1.021	0.831	1.254
		Weighted mode	137	0.036	0.064	0.580	0.580	−0.090	0.162	1.036	0.914	1.176
		MR Egger	146	−0.027	0.036	0.455	0.909	−0.098	0.044	0.973	0.906	1.045
		Weighted median	146	−0.016	0.031	0.596	0.681	−0.077	0.044	0.984	0.926	1.045
LDLC	glaucoma	Inverse variance weighted	146	−0.017	0.026	0.505	0.590	−0.069	0.034	0.983	0.934	1.034
		Simple mode	146	−0.013	0.066	0.841	0.969	−0.142	0.116	0.987	0.867	1.123
		Weighted mode	146	−0.013	0.026	0.612	0.915	−0.064	0.038	0.987	0.938	1.038
		MR Egger	13	0.022	0.045	0.626	0.935	−0.065	0.110	1.023	0.937	1.116
		Weighted median	13	−0.026	0.043	0.545	0.681	−0.110	0.058	0.974	0.896	1.060
LPA	glaucoma	Inverse variance weighted	13	−0.023	0.035	0.516	0.590	−0.092	0.046	0.977	0.913	1.047
		Simple mode	13	−0.035	0.051	0.505	0.969	−0.136	0.065	0.965	0.873	1.068
		Weighted mode	13	−0.030	0.047	0.526	0.915	−0.122	0.061	0.970	0.885	1.063
		MR Egger	130	−0.017	0.091	0.850	0.945	−0.196	0.161	0.983	0.822	1.175
		Weighted median	130	0.039	0.060	0.508	0.799	−0.077	0.156	1.040	0.925	1.169
NAP	glaucoma	Inverse variance weighted	130	−0.034	0.044	0.439	0.935	−0.121	0.052	0.966	0.886	1.054
		Simple mode	130	−0.003	0.146	0.986	0.991	−0.289	0.284	0.997	0.749	1.329
		Weighted mode	130	0.016	0.076	0.836	0.935	−0.133	0.165	1.016	0.875	1.179
		MR Egger	74	−0.061	0.117	0.607	0.945	−0.290	0.169	0.941	0.748	1.184
		Weighted median	74	−0.017	0.072	0.811	0.921	−0.157	0.123	0.983	0.854	1.131
PHOS	glaucoma	Inverse variance weighted	74	−0.028	0.062	0.648	0.985	−0.149	0.093	0.972	0.861	1.097
		Simple mode	74	−0.088	0.149	0.554	0.991	−0.380	0.203	0.915	0.684	1.225
		Weighted mode	74	0.015	0.080	0.850	0.935	−0.142	0.173	1.015	0.867	1.188
		MR Egger	146	0.120	0.058	0.041	0.122	0.006	0.235	1.128	1.006	1.265
		Weighted median	146	0.075	0.044	0.093	0.278	−0.012	0.161	1.077	0.988	1.175
SHBG	glaucoma	Inverse variance weighted	146	0.016	0.035	0.642	0.642	−0.053	0.086	1.017	0.948	1.090
		Simple mode	146	−0.024	0.086	0.778	0.843	−0.194	0.145	0.976	0.824	1.156
		Weighted mode	146	0.079	0.042	0.065	0.196	−0.004	0.162	1.082	0.996	1.176

TBIL	glaucoma	MR Egger	85	0.010	0.051	0.842	0.943	−0.090	0.111	1.010	0.914	1.117
		Weighted median	85	0.062	0.043	0.147	0.344	−0.022	0.145	1.064	0.978	1.157
		Inverse variance weighted	85	−0.004	0.041	0.915	0.984	−0.084	0.075	0.996	0.919	1.078
		Simple mode	85	0.047	0.096	0.622	0.933	−0.140	0.235	1.049	0.869	1.265
		Weighted mode	85	0.018	0.033	0.581	0.745	−0.047	0.084	1.019	0.954	1.087
TES	glaucoma	MR Egger	40	0.033	0.105	0.754	0.754	−0.173	0.239	1.034	0.841	1.270
		Weighted median	40	−0.078	0.077	0.308	0.454	−0.229	0.072	0.925	0.796	1.075
		Inverse variance weighted	40	−0.087	0.063	0.168	0.503	−0.210	0.036	0.917	0.811	1.037
		Simple mode	40	−0.037	0.142	0.796	0.843	−0.316	0.242	0.964	0.729	1.274
		Weighted mode	40	−0.069	0.070	0.337	0.506	−0.207	0.070	0.934	0.813	1.072
TP	glaucoma	MR Egger	111	0.149	0.118	0.207	0.945	−0.081	0.380	1.161	0.922	1.462
		Weighted median	111	0.079	0.061	0.194	0.701	−0.040	0.199	1.083	0.960	1.220
		Inverse variance weighted	111	−0.001	0.061	0.985	0.985	−0.120	0.118	0.999	0.887	1.125
		Simple mode	111	−0.002	0.140	0.991	0.991	−0.276	0.273	0.998	0.759	1.313
		Weighted mode	111	0.047	0.063	0.455	0.849	−0.076	0.170	1.048	0.927	1.185
TRIG	glaucoma	MR Egger	129	0.010	0.058	0.870	0.935	−0.105	0.124	1.010	0.900	1.132
		Weighted median	129	−0.037	0.044	0.399	0.681	−0.124	0.049	0.963	0.883	1.051
		Inverse variance weighted	129	−0.068	0.037	0.066	0.495	−0.140	0.004	0.935	0.870	1.004
		Simple mode	129	−0.018	0.081	0.825	0.969	−0.176	0.140	0.982	0.839	1.151
		Weighted mode	129	−0.018	0.044	0.686	0.915	−0.104	0.069	0.982	0.901	1.071
Urate	glaucoma	MR Egger	115	−0.030	0.113	0.794	0.945	−0.251	0.192	0.971	0.778	1.211
		Weighted median	115	0.030	0.056	0.597	0.821	−0.080	0.140	1.030	0.923	1.150
		Inverse variance weighted	115	0.034	0.052	0.510	0.935	−0.067	0.135	1.035	0.935	1.145
		Simple mode	115	0.054	0.121	0.656	0.991	−0.183	0.291	1.056	0.833	1.338
		Weighted mode	115	0.015	0.061	0.807	0.935	−0.105	0.135	1.015	0.901	1.144
UCR	glaucoma	MR Egger	11	−1.734	1.193	0.180	0.945	−4.072	0.605	0.177	0.017	1.830
		Weighted median	11	0.207	0.254	0.415	0.761	−0.291	0.705	1.230	0.748	2.025
		Inverse variance weighted	11	0.010	0.360	0.978	0.985	−0.695	0.715	1.010	0.499	2.043
		Simple mode	11	0.201	0.387	0.614	0.991	−0.557	0.959	1.223	0.573	2.609
		Weighted mode	11	0.276	0.361	0.463	0.849	−0.432	0.984	1.317	0.649	2.675
URK	glaucoma	MR Egger	4	2.640	5.952	0.701	0.945	−9.027	14.306	14.010	0.000	2.163
		Weighted median	4	−0.922	0.485	0.057	0.630	−1.872	0.028	0.398	0.154	1.029

		Inverse variance weighted	4	−1.299	0.815	0.111	0.572	−2.897	0.298	0.273	0.055	1.347
		Simple mode	4	−0.667	0.737	0.432	0.991	−2.111	0.777	0.513	0.121	2.176
		Weighted mode	4	−0.767	0.644	0.319	0.849	−2.029	0.495	0.464	0.132	1.641
		MR Egger	13	0.103	1.465	0.945	0.945	−2.769	2.976	1.109	0.063	19.600
		Weighted median	13	0.050	0.262	0.849	0.921	−0.464	0.564	1.051	0.629	1.758
URNA	glaucoma	Inverse variance weighted	13	−0.323	0.326	0.322	0.885	−0.963	0.316	0.724	0.382	1.372
		Simple mode	13	0.140	0.385	0.722	0.991	−0.614	0.894	1.150	0.541	2.445
		Weighted mode	13	0.213	0.368	0.573	0.900	−0.508	0.934	1.238	0.602	2.545
		MR Egger	46	−0.002	0.192	0.993	0.993	−0.378	0.375	0.998	0.685	1.454
		Weighted median	46	0.098	0.092	0.286	0.656	−0.082	0.279	1.103	0.921	1.322
VITD	glaucoma	Inverse variance weighted	46	0.035	0.094	0.707	0.922	−0.149	0.220	1.036	0.862	1.246
		Simple mode	46	0.221	0.172	0.206	0.617	−0.117	0.559	1.248	0.890	1.750
		Weighted mode	46	0.049	0.092	0.597	0.935	−0.131	0.229	1.050	0.877	1.258
